# Atmospheric particulates over the northwestern Pacific during the late Holocene: Volcanism, dust, and human perturbation

**DOI:** 10.1126/sciadv.adn3311

**Published:** 2024-10-25

**Authors:** Samuel K. Marx, James Hooper, Tomohisa Irino, Nicola Stromsoe, Krystyna M. Saunders, Osamu Seki, Anthony Dosseto, Andrea Johansen, Quan Hua, Florian Dux, Geraldine Jacobsen, Atun Zawadzki

**Affiliations:** ^1^ Environmental Futures, School of Earth, Atmosphere and Life Sciences, The University of Wollongong, Wollongong, New South Wales, Australia.; ^2^ Earth and Planetary Sciences, School of Science, Hokkaido University, Sapporo, Hokkaido, Japan.; ^3^ Research Institute for the Environment and Livelihoods, Faculty of Science and Technology, Charles Darwin University, Casuarina, Northern Territory, Australia.; ^4^ Australian Nuclear Science and Technology Organisation (ANSTO), Lucas Heights, New South Wales, Australia.; ^5^ Institute of Low Temperature Science, Hokkaido University, Sapporo, Hokkaido, Japan.; ^6^ Wollongong Isotope Geochronology Laboratory, School of Earth, Atmospheric and Life Sciences, The University of Wollongong, Wollongong, New South Wales, Australia.; ^7^ School of Social Science, The University of Queensland, St Lucia, Queensland, Australia.

## Abstract

Mineral aerosols form a key component of Earth’s dynamic biogeochemical systems, yet their composition and mass are variable in time. We reconstruct patterns in mineral aerosol flux from East Asia, the second largest global dust source, in a peat mire in northern Japan. Using geochemical fingerprinting, we show for the past ~3600 years that high but variable tephra flux dominated regional aerosol loads. A human signal was discernible as elevated pollutant metals, along with East Asian mainland dust, identifiable by its geochemical signature. After ~700 years before the present, dust flux increased as the westerly jet intensified and moved south, the summer monsoon strength reduced, and agriculture expanded. From the 20th century, dust flux increased by two times. Attributable largely to human activity, this demarks a major change in aerosol export to the northwestern Pacific with accompanying increases in fluxes for key micronutrients and increased pollution flux by 16 times.

## Teaser

Human activity has markedly modified the sources and chemistry of dust exported from East Asia over the northwest Pacific Ocean.

## INTRODUCTION

Mineral aerosols are a key component of Earth’s climate and biogeochemical systems (*1*, *2*). Dust transport and deposition represents a pathway for nutrient delivery to ecosystems, with dust-derived micronutrients (including Fe, Co, and Zn) understood to be of most importance for remote ecosystems such as the high-nutrient low-chlorophyll ocean basins, including the North Pacific Ocean (*3*–*5*), where they can stimulate primary productivity (PP) (*1*, *6*). Mineral aerosols influence climate directly through their interaction with solar and thermal radiation, and indirectly through their effect on cloud formation and ice albedo (*7*, *8*). Additionally, by stimulating PP, dust further affects climate through the resulting drawdown of atmospheric CO_2_, a process considered to play a key role in regulating climate over millennial timescales (*2*, *3*). The magnitude of these effects depends upon the composition and concentration of mineral aerosol relative to the background climate state and biogeochemical conditions. Reconstructing present-day and past mineral aerosol loads, composition, and sources remains an important task for better understanding Earth system dynamics. Progress has been achieved in characterizing modern-day (*9*, *10*) and even past dust aerosol loads (*11*); however, characterization of the relative significance of different aerosol sources, temporal variability in emissions, and composition remains underexplored, leading to an incomplete understanding of the variability in mineral aerosol composition (*12*).

It is increasingly recognized that present-day mineral aerosol loads and composition have been perturbed by human activity, although the extent of this varies regionally, with key aerosol transport pathways, such as across the northwestern Pacific, poorly characterized (*13*–*15*). To date, greater research attention has been focused on characterizing contemporary anthropogenic aerosols, with studies typically short-term and often conducted over weeks to months and, in rare cases, years (*13*, *14*). Such studies fail to record the onset of anthropogenic effects on mineral aerosols and, similarly, do not record baseline conditions necessary to accurately quantify changes. Examining aerosol emissions over longer temporal scales (centennial to multimillennial scales) allows for a more accurate assessment of the degree of anthropogenic perturbation of dust and toxic metal loads.

The North Pacific Ocean is a key region for interactions between mineral aerosols and other components of Earth’s climate and biogeochemical systems. It is located directly downwind of the second largest global dust source, the deserts of northwestern China, including the Balkhash-Alakol depression, Junggar, Turpan, Tarim, and Qaidam Pendi Basins, the Hexi corridor, Gobi Desert, and Loess plateau. It is also downwind of eastern Chinese dust sources including the North China Plains, Horqin sandy land, and Hulun Buir plain (*10*). The North Pacific Ocean is also bordered by the Pacific Ring of Fire, indicating that volcanic aerosols form an important, but poorly explored component of the regional aerosol load (*16*–*20*). PP in the ocean waters of the north Pacific is limited by the availability of key micronutrients, including Fe, Co, and Zn, suggesting that variability in dust deposition plays a role in regional PP and in subsequent climate and biogeochemical processes (*3*, *6*, *21*). Similarly, the deposition of East Asian–sourced mineral aerosols on the ice sheets/glaciers of North America may influence their stability (*22*). Critically, within the last ~100 years, much of East Asia, most notably China, has undergone major economic and social transformation, which has likely profoundly altered regional mineral aerosols via changing land use and increased industrial activity (*13*–*15*). While a number of studies have previously explored past changes to East Asian mineral aerosols (*11*–*13*, *15*, *16*, *23*–*27*), many of these may capture local rather than regional changes, do not clearly fingerprint/account for differing aerosol sources, or do not clearly capture the onset/magnitude of human-induced change.

**Fig. 1.**
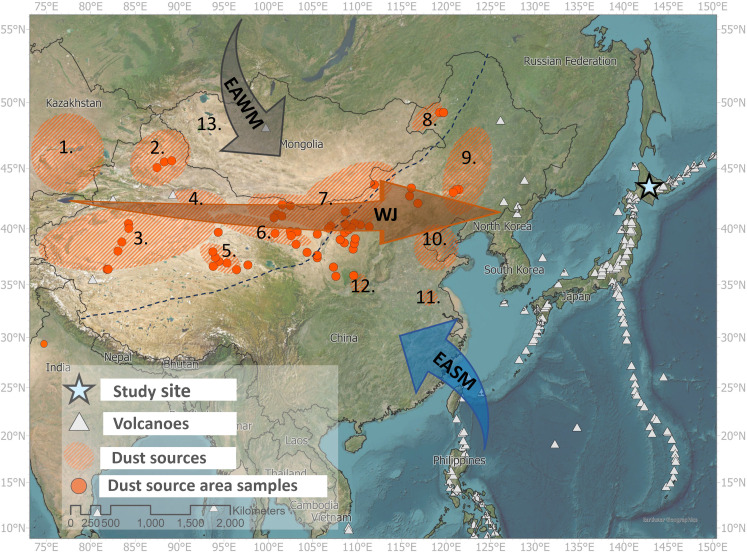
Location map showing the study site, the Ponchubetsudake Mire in the Daisetsuzan Mountains in central Hokkaido, Japan. The locations of samples used to characterize the chemistry of East Asian mineral dust aerosol (*31*, *32*) and volcanoes are indicated, as are major dust-producing regions (*10*). Arrows indicate climate systems considered to influence regional dust transport including the WJ, EASM, and EAWM (*23*, *25*, *42*, *43*). The dashed line represents the modern limit of the reach of the EASM (*50*). The numbers on the figure refer to key dust sources including (1) the Balkhash-Alakol depression, (2) Junggar Pendi, (3) Tarim Pendi, (4) Turpan Pendi, (5) Qaidam Pendi, (6) Hexi corridor, (7) Inner Mongolia deserts, (8) Hulun Buir plain, (9) Horqin sandy land, (10) North China Plains, (11) Hongze and Gaoyou lakes, (12) Tongguan county, and (13) Great Lakes Depression (*10*).

Here, we examine changes in mineral aerosols transported to the northwest Pacific Ocean from East Asia during the late Holocene. Despite past research, the relative contributions of different mineral aerosol sources to the northwest Pacific Ocean remain poorly quantified (*11*, *12*). Similarly, there are few records where the temporal resolution is sufficient to capture the changes in aerosol loads that are assumed to have occurred due to human activity over past centuries, or to examine these changes in the context of longer-term Holocene climate variability (*13*, *14*). We address these knowledge gaps by reconstructing mineral aerosol flux to an ombrotrophic (rainfall/aerosol fed) peat bog (the Ponchubetsudake Mire) in the Daisetsuzan Mountains, central Hokkaido, northern Japan (Fig. 1), a location ideal for characterizing mineral aerosol sources and for examining patterns in mineral aerosol export to the remote northwest Pacific. We use geochemical fingerprinting [using Nd isotopes and conservative trace elements, including the rare earth elements (REEs)] to definitively identify differing mineral aerosol sources, including volcanic aerosols (tephra), mineral dust aerosols from the deserts of northern China, and anthropogenic aerosols, including metal pollutants and nascent dust flux resulting from human land-use intensification. We then track changes in the flux of these differing mineral aerosols over the past three and a half millennia.

## RESULTS

**Fig. 2.**
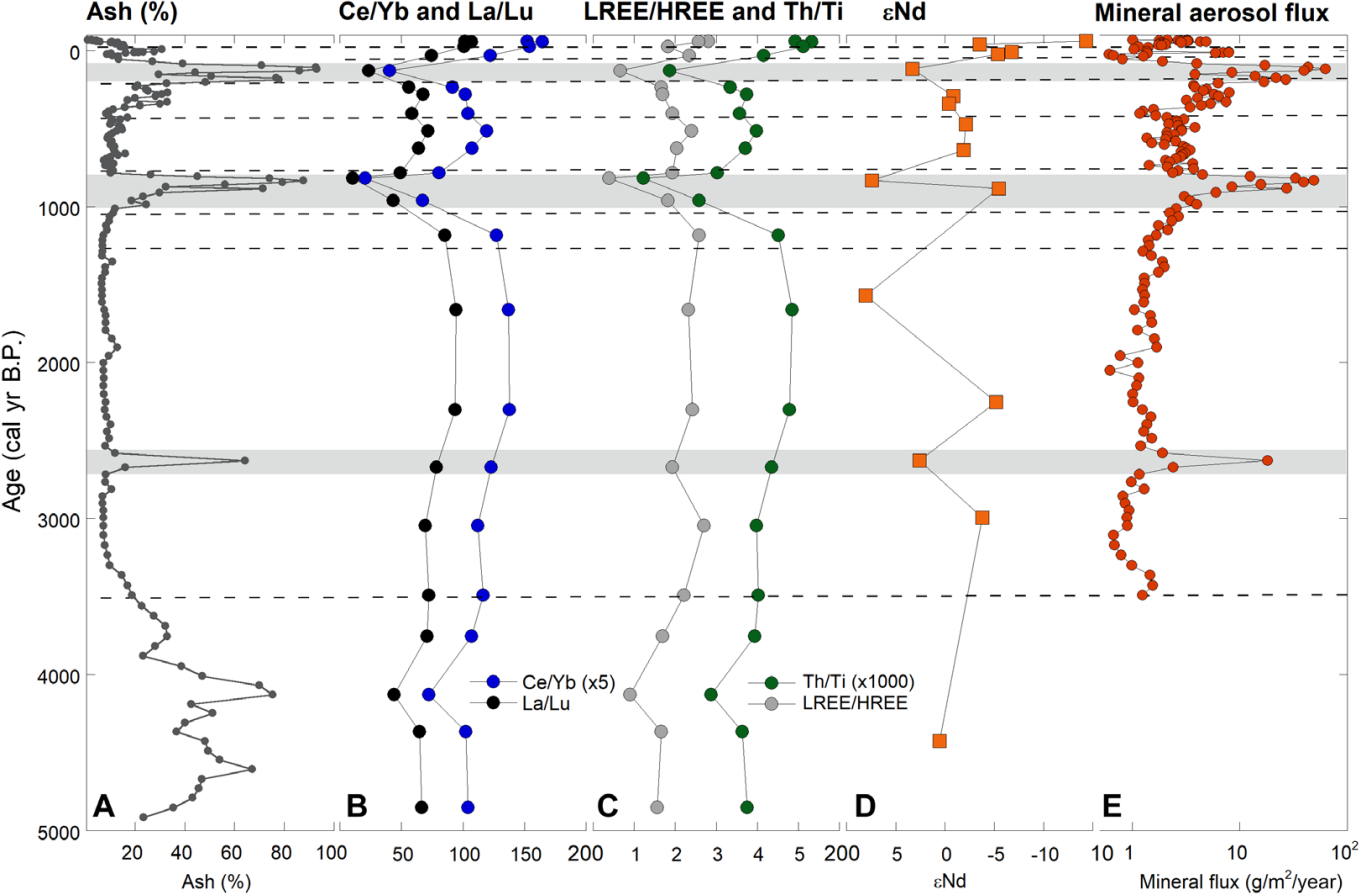
Mineral content and geochemical patterns plotted against time in the Ponchubetsudake Mire core. (**A**) Ash content (% by weight). (**B**) Ce/Yb and La/Lu ratios. (**C**) Th/Ti ratios and the ratio of chondrite-normalized LREE to HREE. (**D**) εNd values. (**E**) Mineral aerosol flux plotted through the ombrotrophic section of the core. Gray bars indicate the location of visible tephra layers in the peat matrix. Dashed lines indicate the locations of change points identified in mineral aerosol flux record. Note that Ce/Yb and Th/Ti ratios are multiplied by 5 and 1000, respectively, for plot clarity.

We collected a 136-cm core from the Ponchubetsudake Mire, Daisetsuzan Mountains, central Hokkaido, Japan. We dated the core using radiocarbon ages and ^210^Pb analyses and developed an age model in the R package rplum (*28*) (fig. S1). All ages from the core increased consistently with depth, demonstrating that the peatland represents a chronologically intact paleo-archive. The basal age indicated that the Ponchubetsudake peatland began developing ~5000 years ago (table S1). We separated the mineral component from the peat matrix within the mire core and used the robust age model to calculate the mineral aerosol flux (Fig. 2). Change-point analysis was undertaken on the mineral aerosol flux, revealing eight distinctive deposition zones in the core (Fig. 2) and implying that aerosol emissions to the northwestern Pacific have been highly dynamic over the past ~3600 years. These deposition zones are generally associated with changes in the geochemical composition of sediment in the core (Fig. 2). Because both Nd isotopes and conservative elements (such as REE) display limited fractionation during mineral aerosol entrainment/emission, transport, deposition, or post-deposition, changes in their abundance indicate changes in the source of mineral aerosol to the study site (*29*).

Results show that the Ponchubetsudake Mire began to record direct atmospheric mineral flux from ~3600 calibrated years before the present (cal yr B.P.), when it became ombrotrophic. That is, the bog switched from receiving runoff-derived alluvium or colluvium to receiving dominantly direct atmospheric deposition. This change is denoted by a reduction in mineral input to the bog, a shift in the geochemistry of bog sediment, including an increase in Th/Ti and La/Lu ratios, and a reduction in the bog growth rate (Fig. 2 and fig. S2).

The early part of the record between 3600 and 1300 cal yr B.P. is characterized by relatively consistent mineral aerosol flux, with a distinctive pattern in conservative element ratios and εNd values, including high ratios of light (LREE) over heavy rare earth elements (HREE), high Th/Ti ratios, and generally positive εNd values (Fig. 2). The greater relative homogeneity in the geochemistry of sediment within the mire before 1300 cal yr B.P. indicates that a consistent and dominant source was supplying mineral aerosol to the atmosphere of the northwestern Pacific. An exception to the generalized pattern occurred at 2627 ± 200 cal yr B.P., where a well-defined tephra (volcanic ash) layer is visible within the peat matrix. This is associated with a shift to positive εNd values and a reduction in the LREE/HREE ratio.

After 1300 cal yr B.P., mineral aerosol flux increased by an average of about four times and became more variable. Similarly, the ratios of Ce/Yb, La/Lu, LREE/HREE, and Th/Ti also became more variable, but at the same time declined, decreasing by an average of 18 to 26% after 1300 cal yr B.P. (26 to 35% if the most recent 100 years of record is excluded). This change is driven by increased deposition of volcanic material, as exemplified by two major tephra deposition events, both evident as broad peaks in the flux record centered at 820 ± 90 and 130 ± 53 cal yr B.P. Like the earlier tephra, these are demarked by distinct geochemical signatures—depletion in LREE/HREE and Th/Ti ratios, and positive excursions in εNd to values of between 3 and 8.

The most striking change in the geochemistry of sediment deposited in the mire occurred within the topmost section of the core, covering the past ~100 years of the record, when the ratios of conservative trace elements increased and εNd values became highly negative. This change is indicative of a highly distinct source of mineral aerosol in the atmosphere of the northwest Pacific, which was not evident during the preceding ~3600 years represented by the Ponchubetsudake Mire.

**Fig. 3.**
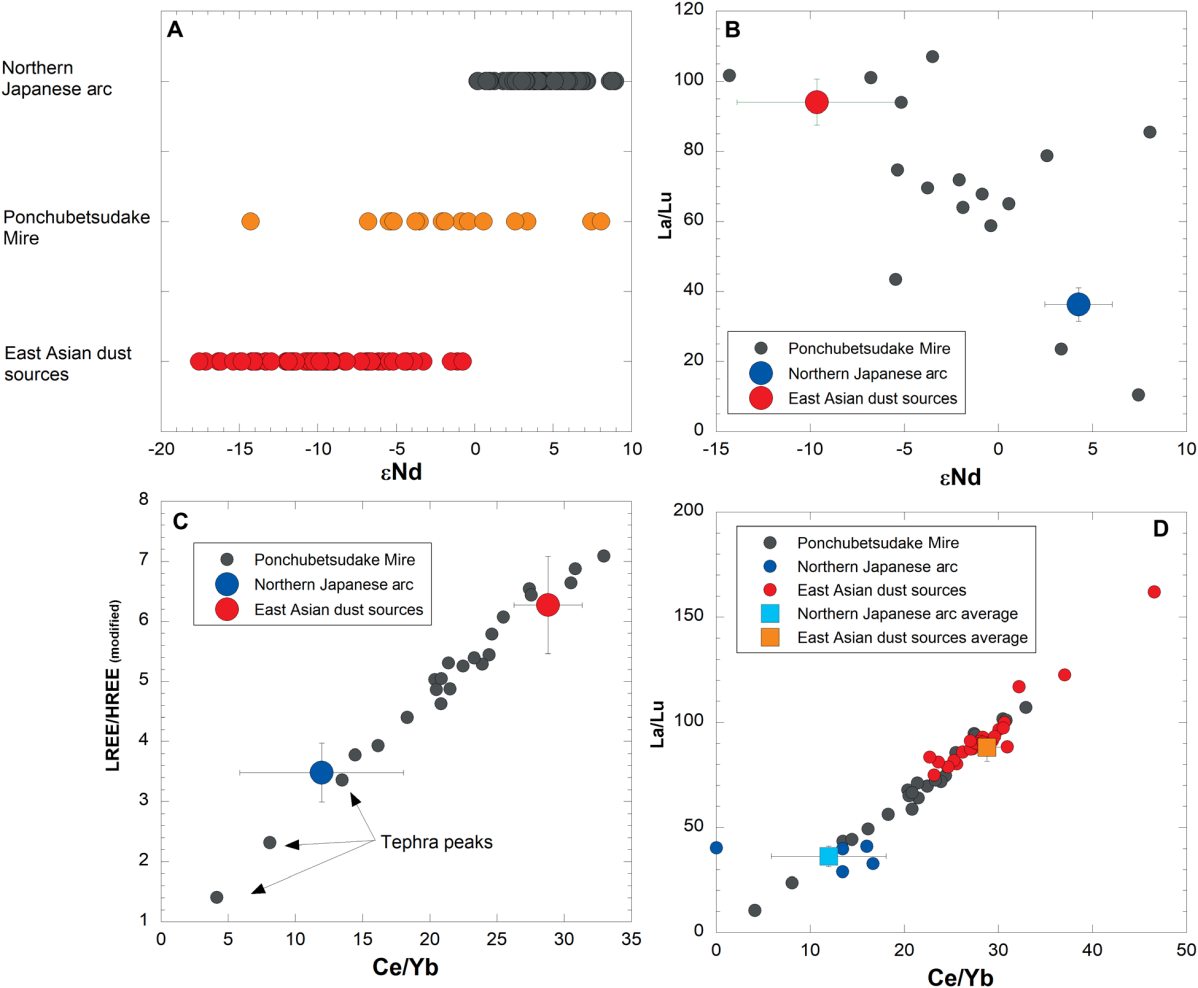
Conservative trace element and εNd composition in the Ponchubetsudake Mire core plotted against samples from East Asian dust sources and local Hokkaido rocks/tephra (*30*–*35*). (**A**) εNd values from sediments in Ponchubetsudake Mire plotted alongside samples representative of potential dust sources in mainland East Asia and rocks from the northern Japanese arc. (**B**) La/Lu versus εNd values in sediments from Ponchubetsudake Mire plotted alongside the average composition of potential dust sources in mainland East Asia and rocks from the northern Japanese arc. (**C**) LLREE/HREE_(modified)_ versus Ce/Yb ratios from sediments in Ponchubetsudake Mire plotted alongside the average composition of potential dust sources in mainland East Asia and rocks from the northern Japanese arc. (**D**) La/Lu versus Ce/Yb ratios from sediments in Ponchubetsudake Mire plotted alongside samples representative of potential dust sources in mainland East Asia and rocks from the northern Japanese arc. Note that as only select REE data are available for Hokkaido rocks, LLREE/HREE_(modified)_ refers to chondrite-normalized (∑ La, Ce, Sm)/(∑ Tb, Yb, Lu).

The range of geochemical variability in conservative geochemical tracers recorded in Ponchubetsudake Mire implies that substantial variability in the sources of “natural” mineral aerosol emitted to the atmosphere of the northwestern Pacific has occurred during the past ~3600 years. Sources of natural aerosols include mineral dust from the East Asian mainland, most notably from the deserts of northwestern China, and volcanic aerosols (tephra) from the array of active volcanoes clustered around the edge of the northwest Pacific Ocean (Fig. 1). These two sources are readily distinguishable from each other using conservative trace element and εNd values, where sediments within dust source areas in mainland East Asia are comprised from continental rocks with negative εNd values and enrichment in LREE (*30*, *31*), while the rocks/sediments of the northwestern Japanese arc (including the island of Hokkaido) are more mantle-derived with positive εNd values and depletion in LREE (*32*–*35*) (Fig. 3). The εNd values of mineral aerosols are extracted from the Ponchubetsudake Mire plot between these endmembers, as is evident in biplots between trace element ratios and εNd values (Fig. 3). Some samples from Ponchubetsudake Mire plot outside the array are defined between the average composition of East Asian dust sources and Japanese arc rocks (Fig. 3C). These represent incursions where one of these sources overwhelmingly dominates the regional mineral aerosol load. They nevertheless fall within the full array of individual trace element signatures for East Asian dust source sediments (Fig. 3D), with their chemistry pointing to their possible origins from specific East Asian dust sources; that is, this dust matches most closely with sediments from the Loess and Tibetan Plateaus, in addition to a potential contribution from the Thar Desert in India, which have the highest trace element ratios (*31*). However, a broader array of source area samples is needed to confirm this. At the other pole of the array, the three major tephra are evident in the bog sediment plot beyond the range of trace element signatures or εNd values analyzed for northwestern Japanese arc rocks. The volcanic eruptions that ejected these tephra display a more highly refined geochemical signature than is currently defined by the relatively limited data characterizing the trace element chemistry of Hokkaido volcanic rocks (*35*). While all the visible tephra deposits are associated with positive εNd values, positive εNd values also occurred at 1570 ± 200 cal yr B.P., without a corresponding spike in mineral flux. This likely represents deposition of locally derived dust, or a local runoff input event.

**Fig. 4.**
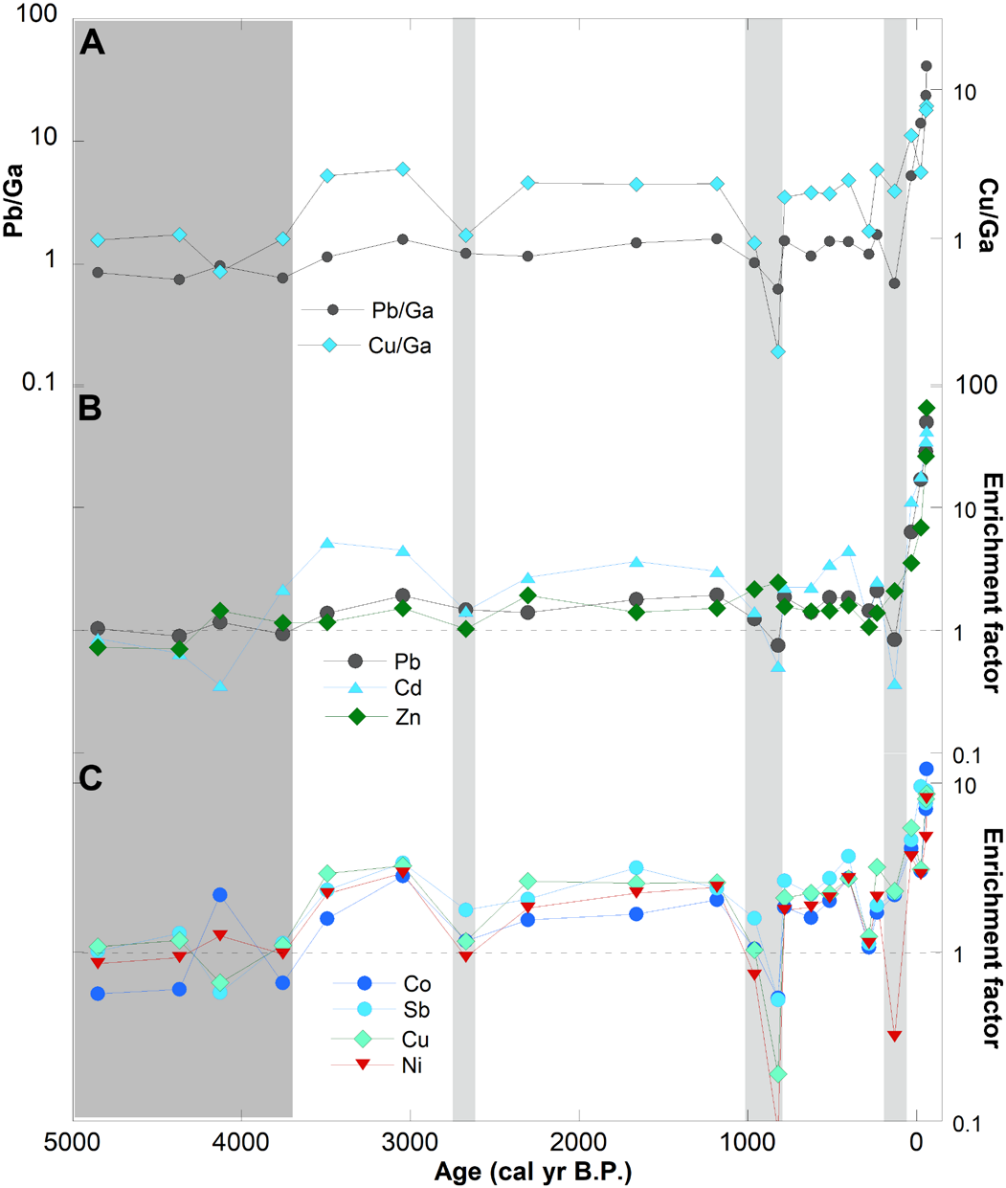
Elements known to be affected by industrial processes plotted through the Ponchubetsudake Mire. (**A**) Pb/Ga and Cu/Ga ratios. The increase in ratio indicates that core samples become enriched in the ombrotrophic section of the core. (**B** and **C**) EFs for various elements calculated against Ga. Dark gray bar indicates the pre-ombrotrophic section of the core; light gray bar indicates the position of tephras visible in the core.

A number of the transition metals, including Cr, Co, Ni, Cu, Zn, Mo, and W, post-transition metals, including In, Sn, Pb, and Tl, and metalloids, including As and Sb, display elevated concentrations in Ponchubetsudake Mire after it became ombrotrophic (from ~3600 cal yr B.P.). This is apparent when these metals are plotted in ratios with highly conservative elements through the mire (e.g., Fig. 4A). This shows that their concentrations increase markedly within the past 100 years. For example, the Pb/Ga ratio in sediment deposited before 3600 cal yr B.P. is 0.83 ± 0.10, similar to the Pb/Ga ratio in average upper continental crust (1.0) (*36*); however, this increases to >40 in top of the mire, indicating a marked increase in Pb deposition (Fig. 4A). All the metals displaying this enrichment pattern have previously been documented to be perturbed in the atmosphere due to human activity (*37*).

The enrichment factor (EF) (or excess metal load) represents the degree to which metal concentrations are elevated in the mire (*15*). We calculated metal EFs for Ponchubetsudake Mire using the pre-ombrotrophic sediments to represent natural background metal concentrations. Results indicate that metals show elevated concentrations from the time the mire became ombrotrophic at ~3600 cal yr B.P. (Fig. 4, B and C), signifying that anthropogenic aerosols represent a significant part of regional mineral aerosol loads from that time. The most noticeable change in EFs occurred within the past 100 years when values increased from ~>1.5 times natural background concentrations to 40 to 60 times natural background concentrations for Pb, Cd, and Zn (Fig. 4B) and >2 to 12 times background for Co, Sb, Cu, and Ni (Fig. 4C) and for Sn, Mo, In, Tl, and W (not plotted).

## DISCUSSION

**Fig. 5.**
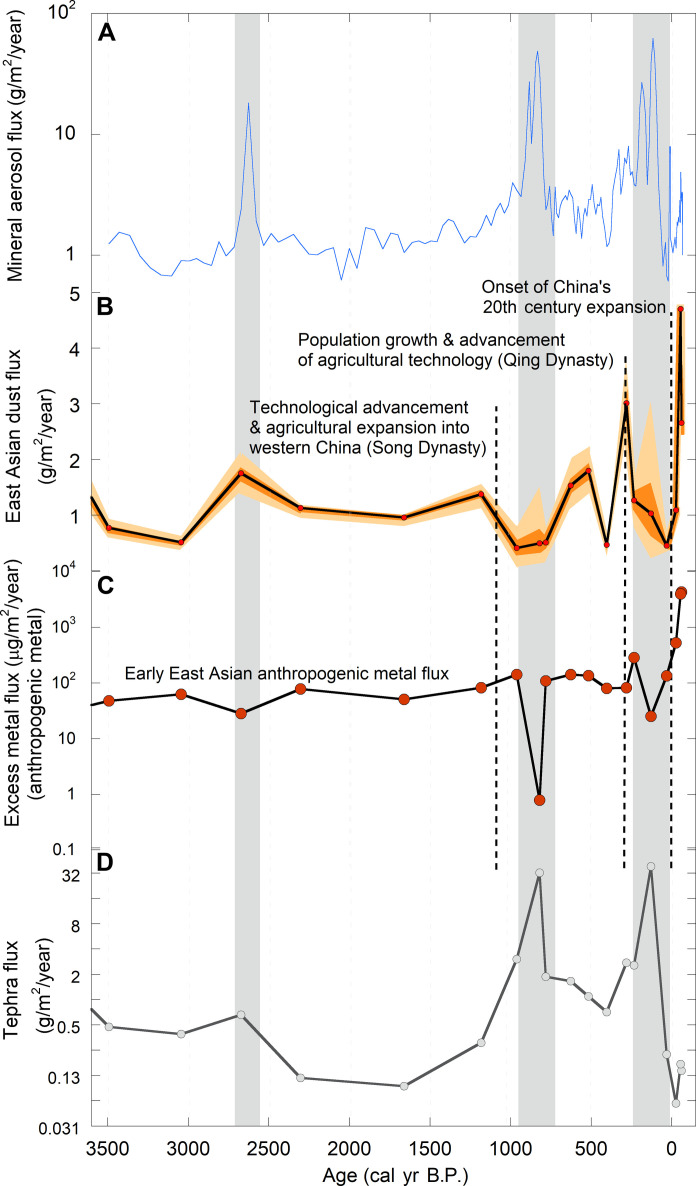
Variability in mineral aerosol flux in Ponchubetsudake Mire. (**A**) Total mineral aerosol flux, (**B**) modeled East Asian mainland dust flux (mean, 1 and 2σ estimates), (**C**) modeled anthropogenic metal flux, and (**D**) modeled tephra flux. Note that total mineral aerosol flux (A) is plotted on a log scale and tephra flux (D) is plotted on a log_2_ scale.

The Ponchubetsudake Mire provides a temporally resolved record in which to examine mineral aerosol loads/sources over Japan and the northwestern Pacific during the late Holocene. Using La/Lu and Ce/Yb ratios from the two main endmember sources of natural mineral aerosols in East Asia (dust from mainland East Asia and volcanic aerosol/local dust), we calculated the time-integrated contribution of mineral aerosols to the mire using a Markov chain Monte Carlo (MCMC) Bayesian framework-based isotope mixing model (Fig. 5). This demonstrated that tephra flux represents the dominant mineral aerosol type in the northwestern Pacific over the past 3700 years, at an average flux of 4.61 ± 11 g m^−2^ year^−1^ (Fig. 5D). Dust flux was lower, but less variable by comparison, averaging 1.37 ± 1.1 g m^−2^ year^−1^, although increased markedly toward the present (Fig. 5D).

Before 1000 cal yr B.P., mainland dust provided 75% of the mineral input to the mire at relatively low fluxes (1.23 ± 0.56 g m^−2^ year^−1^) during a prolonged period of volcanic quiescence (2500 to 1200 cal yr B.P.) (Fig. 5B). Volcanic aerosol (tephra) flux increased after 1000 cal yr B.P., with fluxes reaching 32.4 g m^−2^ year^−1^ at 820 ± 90 cal yr B.P. and 38.7 g m^−2^ year^−1^ at 130 ± 53 cal yr B.P. during major eruptions (Fig. 5D). Tephra deposition at these times completely overwhelmed dust flux. These tephra deposition events occupy broad regions in the core (50 to 150 mm), suggesting that they could represent a series of eruptions, or alternatively, they may also contain redeposition (by the wind) of the presumably large mass of tephra deposited across the Daisetsuzan Mountains and surrounding landscape. The influx of fresh unweathered volcanic sediment into the waters of the northwestern Pacific associated with these eruptions would be expected to have increased the availability of nutrients, particularly Fe, in surface waters (*38*, *39*), with potentially corresponding pulses in PP (*6*, *40*, *41*).

Sometime after ~1000 cal yr B.P., mainland dust flux increased (Fig. 5B). This is apparent after ~700 cal yr B.P. but may have occurred earlier, although the deposition of a substantive tephra, centered at 820 ± 90 cal yr B.P., overwhelms the record at that point. East Asian dust flux increased more substantially toward the present, particularly in the most recent 100 years of the record. Despite the generalized trend of increasing mainland dust flux after 700 cal yr B.P., there is variability in flux rates, including the deposition of a second major tephra centered on 130 ± 53 cal yr B.P., and a notable but short-lived decrease in flux rates at 470 cal yr.

Dust emission from East Asia has been linked to the strength and position of the westerly jet (WJ) which transports dust from source areas in northern China east into the North Pacific, both today and throughout the Quaternary (*23*, *25*, *42*, *43*) (Fig. 1). For example, present-day dust storms are most frequent in spring (*42*, *44*), when the WJ migrates north and intensifies (*45*). Other climate systems have also been invoked to explain past patterns in dust activity. This includes the East Asian winter monsoon (EAWM), which brings cold dry south-easterly winds over northeastern China, with intensification in the EAWM linked to loess production throughout the Quaternary (*24*, *46*–*48*) (Fig. 1). Similarly, dust emission from East Asia is also thought to be affected by the East Asian summer monsoon (EASM), which largely influences rainfall over southern and eastern China, but also within dust source areas in northern China (*23*, *24*, *49*, *50*) (Fig. 1). Our understanding of the effects of these different climate systems for dust emission remains incomplete, although they are known to be interlinked (*45*, *51*, *52*). Variability in their operation has the capacity to influence dust flux recorded in Ponchubetsudake Mire, either directly, by influencing wind strength and plume pathways, or indirectly by influencing antecedent conditions within dust source areas, such as sediment recharge, soil moisture, or vegetation cover over longer timescales (e.g., years to centuries) (*53*–*55*).

**Fig. 6.**
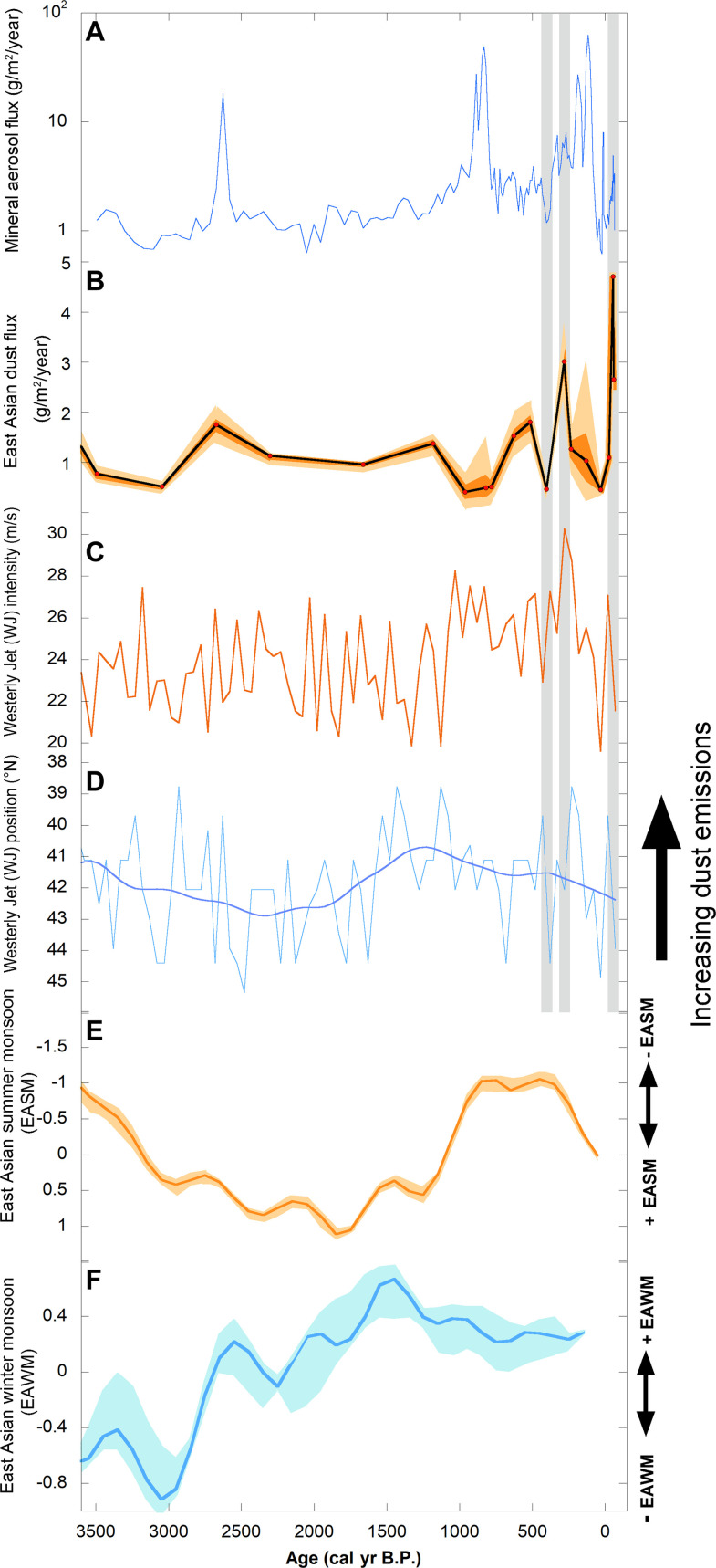
Total mineral flux and East Asian mainland dust flux plotted alongside simulated or composite proxy records of key climate features in East Asia. These include (**A**) total mineral aerosol flux and (**B**) modeled East Asian mainland dust flux (mean, 1 and 2σ estimates) recorded in Ponchubetsudake Mire, (**C**) the simulated intensity and (**D**) position of the Asian WJ (*56*), and composite proxy records of (**E**) the EASM and (**F**) the EAWM (mean and 1σ uncertainty) (*57*). The gray bars show periods within the last 500 cal yr B.P., where East Asian mainland dust flux in Ponchubetsudake Mire either matches or departs from the behavior of the WJ as discussed in the main text. Note that the climate features are all plotted so an upward shift would be expected to result in increased East Asian dust emission (i.e., the WJ position and EASM are plotted with reverse *y* axes). Total mineral aerosol flux (A) is plotted on a log scale.

Irrespective of the exact timing of its onset, the increase in mainland dust deposition in Ponchubetsudake Mire after ~700 cal yr B.P. occurs following a shift in climate that would promote enhanced dust transport (Fig. 6). This includes an intensification and southward positioning of the WJ (*56*) and a corresponding weakening of the EASM (*57*) (Fig. 6). By contrast, the EAWM weakens after 1000 cal yr B.P. (*57*), implying that it was not driving dust flux at this time (Fig. 6F).

The finer structure in the East Asian dust flux in Ponchubetsudake Mire after ~700 cal yr B.P. in the regions of the core not affected by tephra dilution resembles the behavior of inferred WJ intensity and latitudinal position. This includes the decrease in flux at 470 cal yr B.P. and the peak in flux at 270 cal yr B.P. The former occurs as the WJ reduces in intensity and shifts north, while the latter occurs during intensification and southward repositioning of the WJ (Fig. 6, C and D). Despite these linkages, the marked increase in dust flux within the past 100 years occurs as the WJ shifts north and reduces in intensity—conditions that would otherwise limit dust emission (Fig. 6, C and D). However, the onset of enhanced dust flux may match relative changes in the WJ within this period. Increased dust flux also occurs as the EASM intensifies (*57*). This, similarly, would be expected to restrict dust entrainment due to greater advection of moisture into dust sources in northern China (*23*). The lack of an obvious climate link, combined with the magnitude of the dust flux peak, indicates that factors other than climate have contributed to the increased dust flux within the past 100 years, with human activity the most obvious candidate. Human activity been previously argued to be a cause of increased dust in China (*23*, *27*) and increased dust deposition in ice at Mt. Logan, Canada, on the other side of the Pacific Ocean (*13*, *16*). However, it is shown more unequivocally within this study due to the clear geochemical link between the dust in Ponchubetsudake Mire and the deserts of mainland East Asia.

Alongside the changing mineral aerosol load from the East Asian mainland, a distinct nascent anthropogenic aerosol signal in the form of metal pollutants occurred within Ponchubetsudake Mire (Fig. 4). Excess (pollution) metal accumulated from the time Ponchubetsudake Mire became ombrotrophic at ~3600 cal yr B.P., indicating that metallurgy in East Asia was occurring at a scale sufficient to cause regional contamination earlier than previously recognized (*15*, *26*, *58*, *59*). Using the proportion of excess metal (metal enrichment), we calculated anthropogenic metal flux to Ponchubetsudake Mire (Fig. 5C). Excess metal flux increased after 1000 cal yr B.P. (i.e., it averaged 0.67 mg m^−2^ year^−1^ from ~3600 to 1000 cal yr B.P. and 3.5 mg m^−2^ year^−1^ from ~1000 to 250 cal yr B.P.), although, like East Asian dust flux, it was similarly overwhelmed by tephra during volcanic eruptions. This timing of increase is broadly coincident with the beginning of the Song Dynasty, a period of economic expansion and agricultural technological innovation, including in the empires of northwestern China (*60*). The increase in metal flux similarly coincides with the onset of Pb contamination recorded in Alaskan ice fields attributed to East Asian pollution, collectively indicating increasing regional metal contamination (*15*, *16*).

Further increases in excess metal flux are evident in the upper sections of the Ponchubetsudake Mire record, including from ~270 cal yr B.P. (late 1600s CE) during the Qing Dynasty, likely associated with population expansion and the introduction of new agricultural technology, and more markedly from the mid-20th century, after which excess metal flux exceeded 4000 μg m^−2^ year^−1^ (Fig. 5C). The upsurge in anthropogenic metal deposition at the study site is largely attributable to industrial activity in China, which underwent rapid industrial expansion during the second half of the 20th century, with industrial manufacturing increasing from 2.1 to 48.6% of gross domestic product between 1931 and 2008 CE (*61*). Industrial activity in Japan may have also contributed to excess metal loads recorded in Ponchubetsudake Mire; however, the early onset of industrial expansion in Japan (from the 1880s CE) (*62*) does not match the chronology of excess metal flux to Ponchubetsudake Mire. Additionally, the increase in anthropogenic metal flux toward the present in Ponchubetsudake Mire resembles estimated metal emission inventories from China, which exceeded those of Japan after the 1950s CE and peaked in the late 2000s CE, whereas Japan’s emissions decreased after 1970 CE (*63*).

Whereas increased metal flux is directly attributable to human activity, namely, industrial processes, the drivers of dust output can be more complex, with emissions responding to climate (e.g., soil moisture, vegetation cover, and windiness) and geomorphic controls (e.g., sediment production/transport/availability) (*53*). Human activity is known to exacerbate natural dust emissions but remains little explored as a driver of changing dust loads over the Holocene. Despite this, it is becoming increasingly clear that agricultural expansion into arid or semi-arid landscapes is coupled to increased dust generation (*14*, *37*, *64*). This occurs following vegetation clearance, surface disturbance from plowing, introduction of stock animals, and altered hydrological conditions, which individually or in combination expose soils/sediments to the air-stream, increasing their susceptibility to wind entrainment (*13*, *37*).

Given the aforementioned population increases and agricultural expansion within China, the increase in East Asian mainland dust flux in Ponchubetsudake Mire from ~700 cal yr B.P. may be partly attributable to human activity; however, as previously noted, it also occurred when climate conditions are more amenable for dust transport (Fig. 6). The more substantive flux recorded during the 20th century matches the very pronounced expansion of agriculture and industrial activity in China following the Communist Revolution. This included a 32% expansion in agricultural land area between 1961 and 2021 CE, which encompassed the arid/semi-arid regions of northwestern China, the primary dust-producing regions (*10*, *65*), and increased agricultural production, such as a 17% increase in grain production and a 19% increase in grain yield between 1961 and 2011 CE, as well as an approximately 80% increase in grain yield and >90% increase in production of meat and dairy products between 1949 and 2008 CE (*65*). From the 1950s CE northwestern China experienced mass human migration, conversion of native vegetation cover for grazing (*66*, *67*), increased irrigation, and greater mechanization (*68*) [including land abandonment after the Great Leap Forward (*69*)]. As a result, agricultural expansion in northwestern China has been linked anecdotally to pronounced desertification/land degradation, resulting in an increase in dustiness, as recorded in meteorological records and in dust sampling studies (*13*, *66*, *67*, *69*, *70*). Our results indicate that this increase in dust production was of sufficient magnitude to alter regional-scale dust emissions, to a scale whereby it is recorded at sites, like Ponchubetsudake Mire, 5500 to 1500 km distant.

Overall, our data show that volcanic eruptions supplied the largest mass of mineral aerosols to the atmosphere of the northwest Pacific during the late Holocene, with fluxes reaching 30 to 40 g m^−2^ year^−1^ during the largest events, approximately 35 times average background emissions (Fig. 5D). These events likely have profound impacts on downwind biogeochemical systems (*40*, *71*–*73*). The systematic and simultaneous increase in East Asian dust and excess metal flux recorded over the past ~100 years in Ponchubetsudake Mire represents a change in the mass and composition of mineral aerosols in the northwest Pacific. It serves as a striking example of the perturbation of Earth surface processes by human activity, which greatly expanded from the 20th century. The results of this study indicate that dust flux has increased by a factor of >2 within the past 100 years by comparison to the late Holocene, implying a similar magnitude change in dust emissions. The ramping up in dust flux is also associated with enhanced delivery of macro- and micronutrients, with dust-derived Zn, Co, Cu, and P fluxes increasing by 14, 3.4, 2.4, and 2.3 times, within the past ~100 years. The effects of this change on PP remain unquantified, as does the broader effect of the increased dust load on regional climate. We also note the potential for deleterious impacts from the increasing flux of metal pollutants in the region, with Pb, Cd, and Sn fluxes also increasing by between 41 and 7 times over recent decades, with the full impact of this again remaining to be explored.

## MATERIALS AND METHODS

### Sampling site and core processing

The sampling site (43.605°, 142.884°, 1730 m above sea level) consisted of a ~0.24-km^2^ peat bog (the Ponchubetsudake Mire) located east of Ponchubetsudake peak in the central Daisetsuzan Mountains, Hokkaido, Japan. It occupied a gently sloping (approximately 5°) north-facing hill slope located just below the ridge crest. A 136-cm peat core (HOK1A) was extracted from the bog using a Russian D-section corer. In the University of Wollongong, Australia, laboratories, the core was sliced into 0.5-cm samples. Nine samples of peat were selected for radiocarbon dating, spaced at approximately 15-cm intervals, and seven samples were selected for ^210^Pb analysis from within the upper 16 cm of the core. To extract the ash component, subsamples were weighed and dried (at 65°C for 48 hours) and then combusted (at 450°C for 12 hours) before reweighing. The mineral aerosol flux was calculated as the ash component of the core divided by the age range represented by each slice. Twenty of these subsamples evenly spaced through the core were analyzed for trace elements, and 16 were analyzed for Nd isotope (^143^Nd/^144^Nd) composition.

### Geochronological and geochemical analysis

Radiocarbon samples were pretreated using the standard acid-base-acid treatment, combusted, graphitized, and measured by Accelerator Mass Spectrometry (AMS) at the Australian Nuclear Science and Technology Organisation (ANSTO) using the protocols described in (*74*, *75*) (table S1). Lead-210 samples were analyzed by alpha spectrometry using the procedure outlined in (*76*, *77*) (table S2). Ultra-trace element composition was analyzed by solution quadrupole inductively coupled plasma mass spectrometry (ICP-MS) at the University of Melbourne, Australia. Samples were digested in Teflon beakers on a hot plate at 150°C for 48 hours using 1 ml of a 2:1 mixture of concentrated HF-HNO_3_. Following digestion, residual fluorides were converted to nitrates with 0.24 ml of concentrated HNO_3_. Enriched isotopes (^6^Li, ^103^Rh, ^187^Re, ^209^Bi, and ^235^U) were added to correct for internal drift and matrix suppression. Samples were measured on an Agilent 7700x instrument using the protocol of (*78*, *79*). External precision was maintained by repeat analysis of a reference solution every five to eight samples. Laboratory blanks were analyzed with each batch (*n* = ~20) of digested samples, to which results were corrected. The rock standard W2 was used as the calibration standard, while external precision was assessed by analysis of the rock standard JA-2, where standard deviations of REEs <% and were within recommended values (table S3).

Samples were prepared and analyzed for Nd isotopes (^143^Nd/^144^Nd) at the Wollongong Isotope Geochronology Laboratory (WIGL). Up to 50 mg of sample was weighed and digested in a 2:1 mixture of hydrofluoric and nitric acids using Seastar Baseline grade reagents. Following digestion, samples were redissolved in aqua regia (twice if needed) to eliminate fluorides, followed by nitric acid (twice). Samples were then redissolved in 2 M nitric acid before ion exchange chromatography. Neodymium was isolated from the sample matrix using an automated, low-pressure chromatographic system Elemental Scientific prepFAST-MC and a 1-ml Sr-Nd-Pb column (Eichrom) (*80*). The Nd elutions were redissolved in 0.3 M nitric acid. Analysis was performed on a Thermo Fisher Scientific Neptune Plus multicollector ICP-MS at WIGL with an ESI Apex-ST PFA MicroFlow nebulizer (uptake rate of ~0.1 ml min^−1^), an SSI Quartz dual cyclonic spray chamber, jet sample, and H-skimmer cone sample introduction system. Measurements were performed in low-resolution mode. The instrument was tuned at the start of each session with a 200 ppb (parts per billion) Nd solution, and sensitivity for ^142^Nd was typically around 2 V. Masses 140, 142, 143, 144, 145, 146, 148, and 150 were collected in static mode on Faraday cups for 90 cycles, with an integration time of 8.389 s for each cycle. Instrumental mass bias was internally corrected using the measured ^146^Nd/^144^Nd ratio and an exponential law. Masses 147 and 140 were used to correct for the isobaric interference of Sm and Ce, respectively (although only isobaric interference correction for Sm is needed for the ^143^Nd/^144^Nd ratio). The total procedure blank was 60 ng of Nd. Reference materials GSP-2 and JNdi-1 were processed along with the samples and yielded ^146^Nd/^144^Nd ratios within or to error of recommended values (table S4).

### Data analysis

An age model was constructed for the core from the ^14^C and ^210^Pb determinations using rplum, a Bayesian MCMC statistical package in RStudio (*28*) (fig. S1). IntCal20 (*81*) was used to calibrate all the radiocarbon ages, except the upper most radiocarbon sample, which was calibrated using the NH1 bomb radiocarbon data (*82*). Change-point analysis was undertaken on the mineral ash record in the core using the Bayesian regression change package mcp in RStudio (*83*). The contribution of tephra/local dust versus dust sourced from East Asia was estimated using the package SIMMR, a MCMC Bayesian framework endmember-based isotope mixing model, in RStudio (*84*). Although designed as a stable isotope mixing model, SIMMR is readily applicable to modeling sediment provenance using other geochemical systems (*85*). East Asian dust flux was subsequently calculated as the proportion of East Asian dust, determined using SIMMR, within the total mineral flux to the core. Neodymium isotopes (^143^Nd/^144^Nd) are expressed as εNd relative to CHondritic Uniform Reservoir (CHUR) values of (*86*). Anthropogenic metal EFs were calculated relative to the conservative element Ga, from EF = (M/Ga)_s_/(M/Ga)_avgb_, where M denotes a metal whose concentration is suspected of being perturbed by industrial activity, the subscript avgb denotes the average M/Ga ratio in the pre-ombrotrophic section of the core, and s denotes the M/Ga ratio in any sample from the ombrotrophic section of the core. Excess (anthropogenic) metal flux was then derived from the sum of the mass of all excess metal present in core [determined from the concentration of excess metal (M_ex_) in a given sample, i.e., M_ex_ = Sm − (Sm/EF), where Sm is the sum all trace elements in that sample]. Additional data related to this paper may be requested from the authors.

## Supplementary Materials

### This PDF file includes:

Figs. S1 and S2 Tables S1 to S4 References
