# Supplementary material for: Atmospheric particulates over the northwestern Pacific during the late Holocene: Volcanism, dust, and human perturbation
Source: Sci Adv. 2024 Oct 25;10(43):eadn3311. doi: 10.1126/sciadv.adn3311 (PMC12680304; doi:10.1126/sciadv.adn3311)
Supplement: Supplementary file 1 — Figs. S1 and S2 Tables S1 to S4 References [file sciadv.adn3311_sm.pdf]

Science Advances
AAAS

Supplementary Materials for

Atmospheric particulates over the northwestern Pacific during the late Holocene: Volcanism, dust, and human perturbation

Samuel K. Marx *et al.*

Corresponding author: Samuel K. Marx, smarx@uow.edu.au

Sci. Adv. **10**, eadn3311 (2024)
DOI: 10.1126/sciadv.adn3311

This PDF file includes:

Figs. S1 and S2
Tables S1 to S4
References

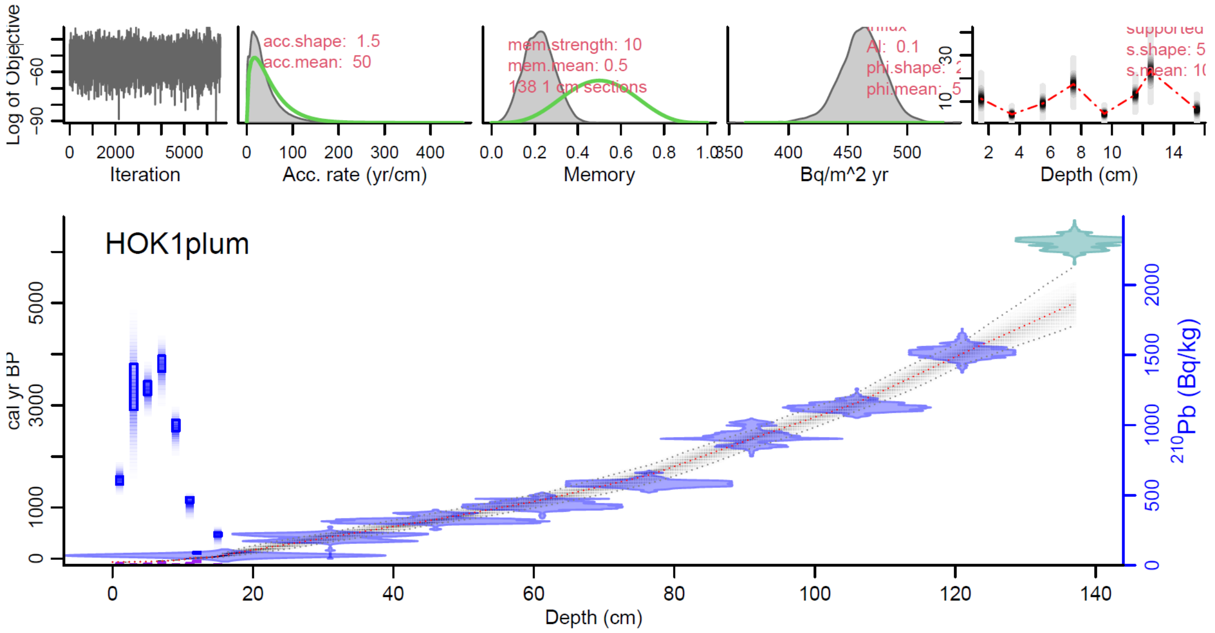

Figure. S1: Age model for the Ponchubetsudake Mire. The age model was developed in the package rplum (22,87). The upper panels display the input parameters and posterior distributions. The bottom panel shows the age model (central dashed red line) and 95% confidence intervals (upper and lower dashed red lines). Light transparent blue/green symbols indicate the calibrated radiocarbon ages and dark blue rectangles indicate the ^{210}Pb profile (Bq/kg) as outlined in Tables S1 and S2.

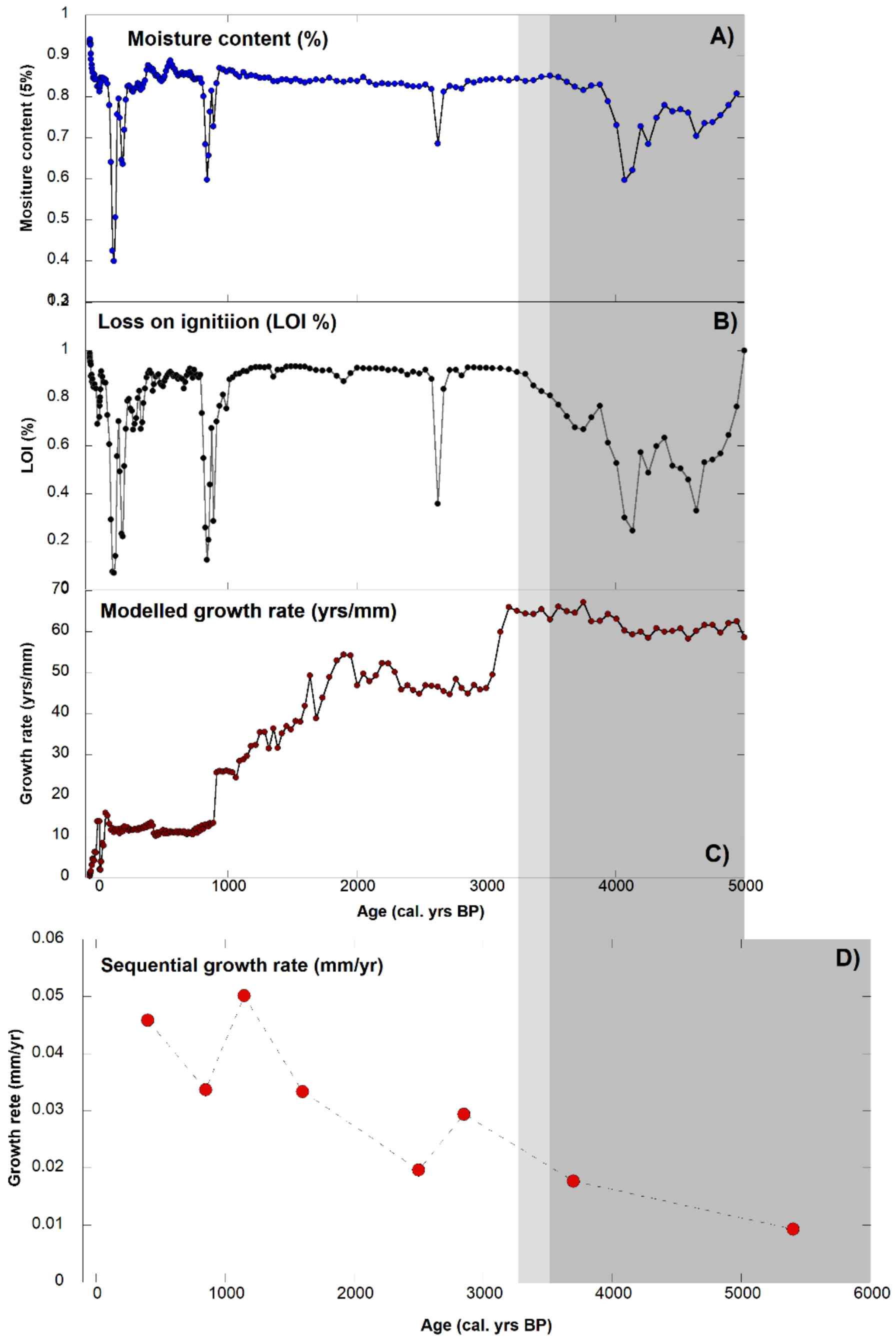

Figure S2. Variability in key parameters within Ponchubetsudake Mire. A) Moisture content, B) loss on ignition, C) modelled peat growth rate, and D) the sequential peat growth rate for the. The modelled growth rate (C) represents the core rate growth in yrs/mm determined from the age model (Fig. S1). The sequential growth rate (D) represents the age difference divided by the depth difference between successive radiocarbon ages and is presented in mm/yr. The lower section of the core before ~3600 cal. yrs BP represents the pre-ombrotrophic portion of the core, where the mineral composition of the peat was dominated by alluvial/colluvial input. It is characterised by low relative moisture content, low relative organic content and a low growth rate, typical of minerogenic peat (29, 88-95). It is denoted by the dark grey shade in Fig. S2. This region of the core is also characterised by a slower growth rate (Fig. S2C & D),

with the lower most radiocarbon age plotting outside the modelled distribution of the other ages (Fig S1). The light grey bar in Fig. S2 denotes the possible transition zone between the ombrotrophic and non-ombrotrophic regions of the core. There are additional troughs in moisture and LOI above ~3600 cal. yrs BP. These represent periods of pronounced tephra flux and are clearly identifiable based on the distinctive geochemical fingerprints, as discussed in detail in the main text and demonstrated in Fig. 2.

Table S1: Radiocarbon analyses and ages.

ANSTO Code	Depth (cm)	$\delta^{13}C$		Percent modern carbon		Conventional Radiocarbon age	
		‰	±1σ	pMC	±1σ	yrs BP	±1σ
OZV635	16	-27.8	0.1	100.46	0.32	Modern	
OZV636	31	-24.9	0.2	96.31	0.36	300	30
OZV637	46	-24.3	0.1	89.23	0.3	915	30
OZV638	61.2	-25.8	0.1	85.9	0.23	1220	25
OZV639	76.4	-26.8	0.3	80.87	0.28	1705	30
OZV640	91	-26.5	0.1	75.02	0.27	2310	30
OZV641	106	-26.1	0.1	70.94	0.27	2760	30
OZV642	121	-24.9	0.1	65.18	0.25	3440	35
OZU857	137	-26.0	0.1	55.65	0.25	4710	40

Table S2: Lead-210 analyses and ages.

ANSTO Code	Depth	Density	Total ^{210}Pb		Supported ^{210}Pb		RPLUM modelled age (yrs cal. BP)		
	cm	g/cm ^{3}	Bq/kg	±1σ	Bq/kg)	±1σ	Median	Min	Max
T386	1.5	0.07	606	27	12.4	3	-65	-65.8	-65.1
T387	3.5	0.07	1272	164	4	1	-62	-63.1	-61.9
T388	5.5	0.08	1264	48	9.3	2	-56.8	-58.5	-54.7
T389	7.5	0.14	1438	57	19.9	3	-44.6	-47.4	-40
T390	9.5	0.16	999	40	4.3	1	-25.6	-30	-17.7
T391	11.5	0.2	466	20	13.6	2	9.9	-1	23.9
T392	12.5	0.18	90	6	28	4	13.8	1.7	30.3
T393	15.5	0.17	221	15	6	2	55.2	28.8	88.6

Table S3: Trace element standards and samples

Name	W-2*			JA2			W2 Preferred values (96)			JA2 Preferred values (96)		
	Digestions = 3 analyses = 3	±2σ	RSD	Digestions = 1 analyses = 2	±2σ	RSD		±2σ	RSD		±2σ	RSD
Li	9,156	121	0.7	28,371	124	0.2	9,210	380	2.1	29,180	560	1.0
Be	617	26	2.1	2,052	22	0.5	672	96	7.1	2,260	190	4.2
P	567,132	21,269	1.9	691,050	6,272	0.5	594,000	52,000	4.4	663,000	1,400	0.1
Ca	77,681,188	393,479	0.3	43,932,412	1,090,795	1.2	77,974,000	428,000	0.3	44,733,000	40,000	0.0
Sc	36,070	211	0.3	18,666	538	1.4	35,860	760	1.1	18,930	340	0.9
Ti	6,354,333	23,997	0.2	3,848,908	83,310	1.1	9,616,000	120,000	0.6	41,014,000	4,200	0.0
V	261,599	2,140	0.4	116,726	2,355	1.0	265,800	5,800	1.1	119,700	2,400	1.0
Cr	92,803	1,196	0.6	425,471	16,885	2.0	92,000	3,200	1.7	424,800	9,300	1.1
Co	44,521	430	0.5	28,768	836	1.5	44,370	1,300	1.5	28,330	970	1.7
Ni	69,998	348	0.2	134,727	2,873	1.1	72,000	2,000	1.4	136,000	2,200	0.8
Cu	102,992	582	0.3	26,882	812	1.5	105,900	3,000	1.4	29,000	1,500	2.6
Zn	76,968	4,051	2.6	62,287	1,684	1.4	77,700	3,200	2.1	64,500	2,300	1.8
Ga	17,425	82	0.2	16,137	292	0.9	17,880	620	1.7	16,850	770	2.3
As	782	143	9.1	338	44	6.5	1,070	320	15.0	710	280	19.7
Rb	19,805	183	0.5	69,806	300	0.2	20,230	540	1.3	69,800	1,300	0.9
Sr	194,852	1,824	0.5	242,231	585	0.1	195,400	3,200	0.8	245,800	3,000	0.6
Y	20,115	112	0.3	15,649	90	0.3	21,820	660	1.5	16,890	580	1.7
Zr	87,909	4,468	2.5	107,135	741	0.3	93,300	2,800	1.5	108,500	2,600	1.2
Nb	7,275	47	0.3	8,846	63	0.4	7,510	300	2.0	9,300	240	1.3
Mo	423	8	0.9	536	5	0.4	465	60	6.5	581	35	3.0
Cd	77	4	2.9	62	6	4.5	74	28	18.9	69	19	13.8
In	65	1	0.5	42	1	0.6	61	8	6.7	37	4	5.0
Sn	1,951	105	2.7	1,715	13	0.4	1,920	240	6.3	1,690	150	4.4
Sb	709	90	6.3	120	3	1.4	809	138	8.5	150	30	10.0
Cs	888	9	0.5	4,757	11	0.1	915	32	1.7	4,780	87	0.9
Ba	169,706	1,778	0.5	307,760	169	0.0	172,800	3,800	1.1	308,400	5,100	0.8
La	10,522	62	0.3	15,553	33	0.1	10,630	240	1.1	15,460	400	1.3
Ce	23,218	123	0.3	32,472	97	0.1	23,210	340	0.7	32,860	850	1.3
Pr	3,026	20	0.3	3,683	2	0.0	3,018	66	1.1	3,691	79	1.1
Nd	12,911	73	0.3	13,984	104	0.4	13,090	240	0.9	14,040	240	0.9
Sm	3,266	30	0.5	3,010	35	0.6	3,300	260	3.9	3,032	43	0.7
Eu	1,094	12	0.5	879	10	0.5	1,091	22	1.0	893	18	1.0
Gd	3,708	4	0.1	2,960	12	0.2	3,713	78	1.1	3,013	85	1.4
Tb	615	3	0.3	471	4	0.4	627	16	1.3	479	8	0.8
Dy	3,808	9	0.1	2,867	9	0.1	3,806	58	0.8	2,851	71	1.2
Ho	803	4	0.2	604	4	0.4	791	12	0.8	591	15	1.3
Er	2,222	2	0.0	1,688	7	0.2	2,208	50	1.1	1,676	31	0.9
Tm	327	1	0.1	256	1	0.2	332	13	1.9	255	7	1.3
Yb	2,058	3	0.1	1,647	27	0.8	2,054	32	0.8	1,645	36	1.1
Lu	301	1	0.2	247	3	0.5	309	7	1.1	255	9	1.8
Hf	2,357	79	1.7	2,758	4	0.1	2,444	82	1.7	2,838	62	1.1
Ta	454	2	0.2	639	1	0.0	489	28	2.9	652	17	1.3
W	240	35	7.3	1,106	24	1.1	290	100	17.2	1,150	30	1.3
Tl	90	1	0.6	328	3	0.4	104	26	12.5	330	13	2.0
Pb	7,531	434	2.9	18,459	240	0.7	7,830	380	2.4	18,880	290	0.8
Bi	24	0	0.7	88	2	1.3	32	22	34.4	92	7	4.0
Th	2,105	56	1.3	4,676	5	0.0	2,179	62	1.4	4,800	110	1.1
U	505	20	2.0	2,243	9	0.2	505	14	1.4	2,182	61	1.4

*Calibration standard

Table S3 Continued

Name	HOK-1A-10	HOK-1A-13	HOK-1A-20	HOK-1A-30	HOK-1A-38	HOK-1A-47	HOK-1A-51	HOK-1A-61	HOK-1A-71	HOK-1A-81	HOK-1A-95	HOK-1A-98
Li	8,660	11,182	15,725	11,999	14,465	12,925	13,130	9,506	9,523	11,633	9,969	10,968
Be	1,024	923	1,650	1,851	590	1,470	1,051	1,373	1,318	1,587	1,704	591
P	18,425,511	10,973,800	8,652,467	8,858,234	546,228	5,317,481	4,176,767	8,928,771	6,450,026	6,448,941	5,464,662	701,190
Ca	85,543,730	28,675,995	30,620,433	55,914,484	47,966,821	37,531,783	24,428,964	99,678,740	63,627,116	67,756,096	80,062,550	24,563,058
Sc	4,379	5,472	12,603	19,715	25,252	19,097	18,097	17,104	15,877	16,526	15,732	17,308
Ti	1,071,976	1,597,490	4,227,287	2,888,649	4,135,771	3,853,852	4,078,899	2,871,681	2,793,026	3,402,331	2,909,096	3,560,636
V	50,273	42,689	103,726	231,754	131,132	571,502	150,199	198,348	118,593	165,903	121,507	24,810
Cr	27,309	33,720	47,104	50,319	9,114	38,486	45,464	38,791	39,943	40,812	29,735	1,727
Co	35,880	23,422	15,665	16,494	12,137	9,006	6,090	11,827	8,253	7,629	7,681	2,487
Ni	44,098	29,268	27,541	27,078	3,255	20,474	11,803	21,696	15,752	16,181	13,538	744
Cu	73,155	76,651	45,844	62,574	36,592	47,808	20,248	33,654	25,943	30,493	24,942	2,538
Zn	1,033,020	470,184	191,712	76,388	62,295	39,142	32,670	37,138	31,672	36,543	34,932	61,016
Ga	9,445	10,607	16,535	12,792	17,727	16,709	18,190	13,763	13,018	15,167	13,257	14,781
As	9,301	8,530	25,836	11,361	8,021	10,611	5,820	13,878	9,063	7,470	8,983	3,308
Rb	232,485	129,662	116,215	58,117	28,444	37,740	42,459	40,118	40,716	53,056	49,982	12,204
Sr	725,737	287,421	339,416	476,789	256,683	343,223	247,610	839,037	572,926	619,497	692,184	199,988
Y	16,531	12,809	17,617	23,076	21,939	28,735	18,784	27,045	24,069	28,649	26,915	35,683
Zr	50,576	53,729	79,415	60,694	85,691	97,140	109,313	68,863	80,093	88,079	90,629	99,934
Nb	3,475	5,472	10,113	6,532	3,528	7,059	7,567	5,848	6,313	7,401	7,902	1,356
Mo	5,108	4,045	3,240	3,246	1,025	2,791	1,621	5,575	3,483	4,028	4,124	1,691
Cd	7,438	6,900	5,587	2,708	122	781	391	1,151	837	634	553	141
In	307	247	244	132	59	111	84	98	80	104	104	86
Sn	6,512	8,036	12,110	2,984	980	2,454	2,519	2,026	1,964	2,384	2,058	1,201
Sb	2,409	2,281	4,480	1,668	1,130	896	579	1,439	1,009	951	994	219
Cs	7,582	11,239	7,663	6,884	3,079	3,511	3,863	3,188	3,488	3,974	3,884	1,158
Ba	1,657,031	767,312	659,856	581,676	358,168	482,131	436,803	568,048	567,644	635,932	616,854	291,309
La	19,033	18,071	26,585	24,761	8,933	24,375	20,558	22,900	24,873	26,558	20,310	6,828
Ce	40,008	38,093	55,132	55,314	20,473	52,820	41,391	54,749	56,261	60,478	44,738	17,813
Pr	4,664	4,443	6,446	6,649	2,689	6,349	4,844	6,363	6,443	6,975	5,356	2,657
Nd	17,629	16,534	23,852	25,497	11,518	25,341	18,201	25,828	25,305	27,695	21,286	12,993
Sm	3,397	3,138	4,653	5,354	2,985	5,635	3,705	5,676	5,279	5,861	4,747	3,998
Eu	709	643	958	1,233	873	1,476	881	1,367	1,212	1,369	1,078	1,261
Gd	3,029	2,641	3,888	4,904	3,416	5,581	3,483	5,535	5,005	5,652	4,773	5,121
Tb	450	394	574	739	593	864	547	842	756	863	768	913
Dy	2,524	2,184	3,303	4,281	3,796	5,149	3,314	4,890	4,426	5,111	4,662	6,106
Ho	517	440	669	863	842	1,089	708	1,013	906	1,051	985	1,387
Er	1,413	1,214	1,854	2,390	2,480	3,050	2,029	2,808	2,517	2,947	2,819	4,105
Tm	211	181	282	355	380	457	310	413	369	438	427	645
Yb	1,312	1,157	1,789	2,266	2,527	2,884	2,032	2,627	2,355	2,814	2,768	4,292
Lu	187	169	263	332	380	434	303	390	346	415	412	655
Hf	1,333	1,442	2,306	1,762	2,542	2,746	3,107	1,938	2,269	2,465	2,536	3,168
Ta	252	398	747	472	245	517	569	411	465	539	537	96
W	1,818	1,931	2,411	1,369	1,074	1,104	1,104	1,056	966	1,103	948	456
Tl	636	818	3,225	2,010	269	1,009	1,027	969	784	920	791	140
Pb	387,593	251,125	231,467	66,930	12,264	28,953	21,787	20,867	19,865	17,573	20,485	9,197
Bi	2,958	2,884	4,561	1,690	85	547	418	609	584	528	678	91
Th	3,014	4,115	7,695	6,771	2,765	6,390	6,918	5,562	6,687	6,937	5,631	1,413
U	1,009	1,241	2,021	1,664	859	1,828	1,945	1,871	1,925	1,895	1,472	494

Table S3 Continued

Name	HOK-1A-106	HOK-1A-114	HOK-1A-127	HOK-1A-140	HOK-1A-148	HOK-1A-171	HOK-1A-178	HOK-1A-182	HOK-1A-188	HOK-1A-192	HOK-1A-200
Li	13,590	10,285	11,290	12,997	11,744	9,878	8,261	15,802	13,910	14,194	14,155
Be	2,497	1,351	1,297	1,457	1,098	1,566	1,011	1,138	968	1,121	1,675
P	2,460,891	6,192,578	5,686,981	7,600,092	4,720,907	6,586,496	4,598,043	4,283,526	1,391,947	4,280,684	3,231,381
Ca	51,059,759	119,922,323	102,349,938	88,252,792	48,595,308	84,298,554	32,499,013	20,470,397	34,990,620	16,618,721	18,523,376
Sc	17,726	10,044	10,460	12,917	18,397	12,619	15,252	18,302	26,090	22,234	19,687
Ti	3,551,987	2,664,338	3,120,243	3,529,679	3,735,075	2,751,248	2,749,954	4,804,121	7,636,441	4,657,655	4,995,662
V	107,394	85,791	89,389	106,252	134,982	174,789	165,552	193,181	183,343	232,731	228,276
Cr	16,134	38,925	43,884	50,331	39,156	41,785	39,015	55,052	79,904	54,135	46,058
Co	6,026	7,166	6,513	7,319	6,501	11,067	5,568	4,707	16,525	3,988	3,802
Ni	7,770	15,584	15,986	15,798	9,601	21,215	14,434	12,864	17,435	11,335	10,541
Cu	17,047	25,857	28,373	35,433	18,515	36,342	29,447	22,504	14,332	22,228	20,670
Zn	66,853	28,489	29,303	48,802	30,699	31,946	21,994	44,181	58,650	25,089	25,939
Ga	18,395	11,162	12,371	15,013	17,751	12,506	11,229	22,785	24,138	21,080	21,305
As	9,099	8,351	14,569	12,747	13,721	31,175	16,340	11,349	7,823	12,850	16,830
Rb	43,313	33,045	40,057	55,391	53,957	51,259	50,633	78,490	46,902	54,636	65,512
Sr	409,721	1,083,626	914,533	747,097	470,535	741,512	299,971	230,526	260,512	178,895	209,525
Y	42,455	14,252	15,717	19,015	18,388	27,674	22,598	21,309	19,504	24,398	25,946
Zr	242,801	68,893	75,251	83,824	75,908	76,118	72,822	122,777	128,003	124,716	131,297
Nb	23,734	8,587	9,509	10,635	7,662	8,216	6,139	9,290	6,850	7,638	8,759
Mo	4,419	3,299	3,732	4,599	4,479	7,933	5,934	4,530	2,248	8,772	6,457
Cd	488	630	839	758	476	1,045	1,093	916	161	254	341
In	114	78	85	109	96	122	124	106	73	100	91
Sn	3,053	2,374	2,911	3,213	2,477	2,582	2,341	3,080	2,629	2,615	2,994
Sb	826	754	1,108	877	893	1,197	740	729	395	772	611
Cs	2,267	3,233	3,451	4,075	3,268	3,974	5,347	8,431	3,018	6,963	8,642
Ba	458,312	979,424	754,425	720,537	554,109	592,749	341,948	455,694	392,776	378,396	402,139
La	29,028	19,855	23,863	27,877	22,759	29,185	24,455	25,270	15,263	24,967	27,678
Ce	61,757	39,510	47,092	56,262	48,553	62,526	52,920	50,506	32,944	53,001	57,960
Pr	7,421	4,566	5,417	6,519	5,752	7,401	6,152	5,894	3,896	6,210	6,605
Nd	29,452	17,034	19,980	24,114	21,975	28,815	24,338	22,294	15,344	24,192	25,455
Sm	7,072	3,313	3,803	4,632	4,466	5,850	4,988	4,443	3,459	5,139	5,057
Eu	1,258	700	797	979	1,172	1,374	1,236	1,044	1,062	1,237	1,205
Gd	7,376	2,845	3,223	3,950	3,862	5,398	4,643	4,014	3,446	4,833	4,763
Tb	1,249	439	487	605	592	813	693	622	567	764	743
Dy	7,757	2,588	2,840	3,504	3,474	4,809	4,119	3,792	3,541	4,601	4,532
Ho	1,641	536	594	729	711	1,011	865	815	765	966	975
Er	4,656	1,541	1,690	2,033	2,003	2,871	2,415	2,356	2,219	2,707	2,796
Tm	705	237	260	310	308	434	358	362	342	399	425
Yb	4,584	1,551	1,718	2,042	1,972	2,784	2,271	2,361	2,281	2,586	2,780
Lu	668	232	252	297	289	420	337	356	345	384	416
Hf	6,258	1,915	2,115	2,334	2,178	2,107	2,076	3,395	3,491	3,412	3,559
Ta	1,373	618	708	785	566	601	448	715	532	583	665
W	1,143	1,135	1,358	1,484	1,166	1,247	1,070	1,444	1,037	1,327	1,512
Tl	443	430	551	672	655	554	518	670	744	585	941
Pb	18,784	17,887	18,306	17,286	21,573	19,809	12,755	17,531	23,210	15,628	18,114
Bi	429	558	916	979	1,761	1,325	901	839	941	762	1,036
Th	6,492	6,890	7,233	8,552	7,239	7,442	6,097	8,156	6,933	7,745	7,841
U	1,599	1,817	2,069	2,355	1,816	2,008	2,001	2,535	2,103	2,508	2,672

Table S4: Nd isotope standards and samples

Name	$^{143}Nd/^{144}Nd$	±2σ	εNd*
GSP-2	0.511391	0.000004	
JNdi-1 (n =13)	0.512121	0.000015	
GSP-2 recommended values (97)	0.511368	0.000011	
JNdi-1 recommended values (98)	0.512115	0.000007	
HOK-1A/08	0.511900	0.000065	-14.285
HOK-1A/17	0.512453	0.000044	-3.495
HOK-1A/24	0.512285	0.000016	-6.779
HOK-1A/29	0.512358	0.000038	-5.348
HOK-1A/37	0.512803	0.000018	3.345
HOK-1A/52	0.512588	0.000014	-0.860
HOK-1A/56	0.512612	0.000012	-0.392
HOK-1A/67	0.512525	0.000011	-2.082
HOK-1A/82	0.512535	0.000009	-1.886
HOK-1A/99	0.513014	0.000014	7.446
HOK-1A/103	0.512352	0.000015	-5.457
HOK-1A/125	0.513045	0.000017	8.054
HOK-1A/139	0.512368	0.000011	-5.156
HOK-1A/147	0.512764	0.000016	2.580
HOK-1A/170	0.512440	0.000010	-3.754
HOK-1A/193	0.512661	0.000012	0.565
HOK-1A/193 - replicate	0.512663	0.000010	0.608

*Calculated relative to CHUR (96)

REFERENCES AND NOTES

1. T. D. Jickells, Z. S. An, K. K. Andersen, A. R. Baker, G. Bergametti, N. Brooks, J. J. Cao, P. W. Boyd, R. A. Duce, K. A. Hunter, H. Kawahata, N. Kubilay, J. laRoche, P. S. Liss, N. Mahowald, J. M. Prospero, A. J. Ridgwell, I. Tegen, R. Torres, Global iron connections between desert dust, ocean biogeochemistry, and climate. *Science* **308**, 67–71 (2005).

2. B. A. Maher, J. M. Prospero, D. Mackie, D. Gaiero, P. P. Hesse, Y. Balkanski, Global connections between aeolian dust, climate and ocean biogeochemistry at the present day and at the last glacial maximum. *Earth Sci. Rev.* **99**, 61–97 (2010).

3. J. Hooper, P. Mayewski, S. Marx, S. Henson, M. Potocki, S. Sneed, M. Handley, S. Gasso, M. Fischer, K. Saunders, Examining links between dust deposition and phytoplankton response using ice cores. *Aeolian Res.* **36**, 45–60 (2019).

4. C. Zhang, J. He, X. Yao, Y. Mu, X. Guo, X. Ding, Y. Yu, J. Shi, H. Gao, Dynamics of phytoplankton and nutrient uptake following dust additions in the northwest Pacific. *Sci. Total Environ.* **739**, 139999 (2020).

5. J. Zan, B. A. Maher, T. Yamazaki, X. Fang, W. Han, J. Kang, Z. Hu, Mid-Pleistocene links between Asian dust, Tibetan glaciers, and Pacific iron fertilization. *Proc. Natl. Acad. Sci. U.S.A.* **120**, e2304773120 (2023).

6. C. M. Moore, M. M. Mills, K. R. Arrigo, I. Berman-Frank, L. Bopp, P. W. Boyd, E. D. Galbraith, R. J. Geider, C. Guieu, S. L. Jaccard, T. D. Jickells, J. La Roche, T. M. Lenton, N. M. Mahowald, E. Maranon, I. Marinov, J. K. Moore, T. Nakatsuka, A. Oschlies, M. A. Saito, T. F. Thingstad, A. Tsuda, O. Ulloa, Processes and patterns of oceanic nutrient limitation. *Nat. Geosci.* **6**, 701–710 (2013).

7. N. Huneeus, M. Schulz, Y. Balkanski, J. Griesfeller, J. Prospero, S. Kinne, S. Bauer, O. Boucher, M. Chin, F. Dentener, T. Diehl, R. Easter, D. Fillmore, S. Ghan, P. Ginoux, A. Grini, L. Horowitz, D. Koch, M. C. Krol, W. Landing, Global dust model intercomparison in AeroCom phase I. *Atmos. Chem. Phys.* **11**, 7781–7816 (2011).

8. J. F. Kok, T. Storelvmo, V. A. Karydis, A. A. Adebiyi, N. M. Mahowald, A. T. Evan, C. He, D. M. Leung, Mineral dust aerosol impacts on global climate and climate change. *Nat. Rev. Earth Environ.* **4**, 71–86 (2023).

9. A. Chappell, N. P. Webb, M. Hennen, K. Schepanski, P. Ciais, Y. Balkanski, C. S. Zender, I. Tegen, Z. Zeng, D. Tong, B. Baker, M. Ekström, M. Baddock, F. D. Eckardt, T. Kandakji, J. A. Lee, M. Nobakht, J. von Holdt, J. F. Leys, Satellites reveal Earth's seasonally shifting dust emission sources. *Sci. Total Environ.* **883**, 163452 (2023).

10. P. Ginoux, J. M. Prospero, T. E. Gill, N. C. Hsu, M. Zhao, Global-scale attribution of anthropogenic and natural dust sources and their emission rates based on MODIS Deep Blue aerosol products. *Rev. Geophys.* **50**, RG3005 (2012).

11. S. Albani, N. M. Mahowald, G. Winckler, R. F. Anderson, L. I. Bradtmiller, B. Delmonte, R. François, M. Goman, N. G. Heavens, P. P. Hesse, S. A. Hovan, K. E. Kohfeld, H. Lu, V. Maggi, J. A. Mason, P. A. Mayewski, D. McGee, X. Miao, B. L. Otto-Bliesner, A. T. Perry, Twelve thousand years of dust: The Holocene global dust cycle constrained by natural archives. *Clim. Past* **11**, 869–903 (2015).

12. J. T. Abell, G. Winckler, A. Pullen, C. W. Kinsley, P. A. Kapp, J. L. Middleton, F. J. Pavia, D. McGee, H. L. Ford, M. E. Raymo, Evaluating the drivers of quaternary dust fluxes to the western North Pacific: East Asian dustiness and Northern Hemisphere gustiness. *Paleoceanogr. Paleoclimatol.* **38**, e2022PA004571 (2023).

13. J. Hooper, S. Marx, A global doubling of dust emissions during the Anthropocene? *Global Planet. Change* **169**, 70–91 (2018).

14. N. P. Webb, C. Pierre, Quantifying anthropogenic dust emissions. *Earths Future* **6**, 286–295 (2018).

15. S. K. Marx, S. Rashid, N. Stromsoe, Global-scale patterns in anthropogenic Pb contaimnination reconstructed from natural archives. *Environ. Pollut.* **213**, 283–298 (2016).

16. E. Osterberg, P. Mayewski, K. Kreutz, D. Fisher, M. Handley, S. Sneed, C. Zdanowicz, J. Zheng, M. Demuth, M. Waskiewicz, J. Bourgeois, Ice core record of rising lead pollution in the North Pacific atmosphere. *Geophys. Res. Lett.* **35**, L05810 (2008).

17. Y. Hayakawa, Catalog of volcanic eruptions during the past 2, 000 years in Japan. *J. Geogr.* **108**, 472–488 (1999).

18. V. C. Smith, R. A. Staff, S. P. E. Blockley, C. Bronk Ramsey, T. Nakagawa, D. F. Mark, K. Takemura, T. Danhara, Suigetsu 2006 Project Members, Identification and correlation of visible tephras in the Lake Suigetsu SG06 sedimentary archive, Japan: Chronostratigraphic markers for synchronising of east Asian/west Pacific palaeoclimatic records across the last 150 ka. *Quat. Sci. Rev.* **67**, 121–137 (2013).

19. H. Mackay, P. D. M. Hughes, B. J. L. Jensen, P. G. Langdon, S. D. F. Pyne-O'Donnell, G. Plunkett, D. G. Froese, S. Coulter, J. E. Gardner, A mid to late Holocene cryptotephra framework from eastern North America. *Quat. Sci. Rev.* **132**, 101–113 (2016).

20. K. Aoki, Revised age and distribution of ca. 87ka Aso-4 tephra based on new evidence from the northwest Pacific Ocean. *Quat. Int.* **178**, 100–118 (2008).

21. P. Pinedo-González, N. J. Hawco, R. M. Bundy, E. V. Armbrust, M. J. Follows, B. B. Cael, A. E. White, S. Ferrón, D. M. Karl, S. G. John, Anthropogenic Asian aerosols provide Fe to the North Pacific Ocean. *Proc. Natl. Acad. Sci. U.S.A.* **117**, 27862–27868 (2020).

22. J. F. Kok, D. S. Ward, N. M. Mahowald, A. T. Evan, Global and regional importance of the direct dust-climate feedback. *Nat. Commun.* **9**, 241 (2018).

23. S. Pratte, K. Bao, C. Li, W. Zhang, G. Le Roux, G. Li, F. De Vleeschouwer, East Asian monsoon and westerly jet driven changes in climate and surface conditions in the NE drylands of China since the Late Pleistocene. *Quat. Sci. Rev.* **331**, 108637 (2024).

24. F. An, H. Ma, H. Wei, Z. Lai, Distinguishing aeolian signature from lacustrine sediments of the Qaidam Basin in northeastern Qinghai-Tibetan Plateau and its palaeoclimatic implications. *Aeolian Res.* **4**, 17–30 (2012).

25. K. Nagashima, R. Tada, A. Tani, Y. Sun, Y. Isozaki, S. Toyoda, H. Hasegawa, Millennial-scale oscillations of the westerly jet path during the last glacial period. *J. Asian Earth Sci.* **40**, 1214–1220 (2011).

26. C. S. L. Lee, S. H. Qi, G. Zhang, C. L. Luo, L. Y. L. Zhao, X. D. Li, Seven thousand years of records on the mining and utilization of metals from lake sediments in central China. *Environ. Sci. Tech.* **42**, 4732–4738 (2008).

27. K. Bao, W. Xing, X. Yu, H. Zhao, N. McLaughlin, X. Lu, G. Wang, Recent atmospheric dust deposition in an ombrotrophic peat bog in Great Hinggan Mountain, Northeast China. *Sci. Total Environ.* **431**, 33–45 (2012).

28. M. Blaauw, J. A. Chisten, M. A. Aquino Lopez. rplum: Bayesian age-depth modelling of cores dated by Pb-210 (2022); https://CRAN.R-project.org/package=rplum.

29. S. K. Marx, H. A. McGowan, B. S. Kamber, Long-range dust transport from eastern Australia: A proxy for Holocene aridity and ENSO-induced climate variability. *Earth Planet. Sci. Lett.* **282**, 167–177 (2009).

30. J. Chen, G. Li, J. Yang, W. Rao, H. Lu, W. Balsam, Y. Sun, J. Ji, Nd and Sr isotopic characteristics of Chinese deserts: Implications for the provenances of Asian dust. *Geochim. Cosmochim. Acta* **71**, 3904–3914 (2007).

31. M. Ferrat, D. J. Weiss, S. Strekopytov, S. Dong, H. Chen, J. Najorka, Y. Sun, S. Gupta, R. Tada, R. Sinha, Improved provenance tracing of Asian dust sources using rare earth elements and selected trace elements for palaeomonsoon studies on the eastern Tibetan Plateau. *Geochim. Cosmochim. Acta* **75**, 6374–6399 (2011).

32. K. Shuto, Y. Hirahara, H. Ishimoto, A. Aoki, A. Jinbo, Y. Goto, Sr and Nd isotopic compositions of the magma source beneath north Hokkaido, Japan: Comparison with the back-arc side in the NE Japan arc. *J. Volcanol. Geotherm. Res.* **134**, 57–75 (2004).

33. K. Takanashi, K. Shuto, M. Sato, Origin of Late Paleogene to Neogene basalts and associated coeval felsic volcanic rocks in Southwest Hokkaido, northern NE Japan arc: Constraints from Sr and Nd isotopes and major- and trace-element chemistry. *Lithos* **125**, 368–392 (2011).

34. K. Takanashi, Y. Kakihara, H. Ishimoto, K. Shuto, Melting of crustal rocks as a possible origin for Middle Miocene to Quaternary rhyolites of northeast Hokkaido, Japan: Constraints from Sr and Nd isotopes and major- and trace-element chemistry. *J. Volcanol. Geotherm. Res.* **221-222**, 52–70 (2012).

35. Y. Masuda, S. Nishimura, T. Ikeda, Y. Katsui, Rare-earth and trace elements in the Quaternary volcanic rocks of Hokkaido, Japan. *Chem. Geol.* **15**, 251–271 (1975).

36. S. M. McLennan, Relationships between the trace element composition of sedimentary rocks and upper continental crust. *Geochem. Geophys. Geosyst.* **2**, (2001).

37. S. K. Marx, H. A. Mc Gowan, B. S. Kamber, J. M. Knight, J. Denholm, A. Zawadzki, Unprecedented wind erosion and perturbation of surface geochemistry marks the Anthropocene in Australia. *J. Geophys. Res. Earth Surface* **119**, 45–61 (2014).

38. Z. Shi, M. D. Krom, S. Bonneville, A. R. Baker, C. Bristow, N. Drake, G. Mann, K. Carslaw, J. B. McQuaid, T. Jickells, L. G. Benning, Influence of chemical weathering and aging of iron oxides on the potential iron solubility of Saharan dust during simulated atmospheric processing. *Global Biogeochem. Cycles* **25**, GB2010 (2011).

39. M. G. Weinbauer, B. Guinot, C. Migon, F. Malfatti, X. Mari, Skyfall—Neglected roles of volcano ash and black carbon rich aerosols for microbial plankton in the ocean. *J. Plankton Res.* **39**, 187–198 (2017).

40. J. Longman, M. R. Palmer, T. M. Gernon, H. R. Manners, The role of tephra in enhancing organic carbon preservation in marine sediments. *Earth Sci. Rev.* **192**, 480–490 (2019).

41. E. P. Achterberg, C. M. Moore, S. A. Henson, S. Steigenberger, A. Stohl, S. Eckhardt, L. C. Avendano, M. Cassidy, D. Hembury, J. K. Klar, M. I. Lucas, A. I. Macey, C. M. Marsay, T. J.

Ryan-Keogh, Natural iron fertilization by the Eyjafjallajökull volcanic eruption. *Geophys. Res. Lett.* **40**, 921–926 (2013).

42. Y. Shao, C. H. Dong, A review on East Asian dust storm climate, modelling and monitoring. *Global Planet. Change* **52**, 1–22 (2006).

43. J. Guo, M. Lou, Y. Miao, Y. Wang, Z. Zeng, H. Liu, J. He, H. Xu, F. Wang, M. Min, P. Zhai, Trans-Pacific transport of dust aerosols from East Asia: Insights gained from multiple observations and modeling. *Environ. Pollut.* **230**, 1030–1039 (2017).

44. G. Roe, On the interpretation of Chinese loess as a paleoclimate indicator. *Quatern. Res.* **71**, 150–161 (2009).

45. R. Schiemann, D. Lüthi, C. Schär, Seasonality and interannual variability of the westerly jet in the Tibetan Plateau region. *J. Climate* **22**, 2940–2957 (2009).

46. Y. Sun, S. C. Clemens, Z. An, Z. Yu, Astronomical timescale and palaeoclimatic implication of stacked 3.6-Myr monsoon records from the Chinese Loess Plateau. *Quat. Sci. Rev.* **25**, 33–48 (2006).

47. Z. An, T. Liu, Y. Lu, S. C. Porter, G. Kukla, X. Wu, Y. Hua, The long-term paleomonsoon variation recorded by the loess-paleosol sequence in Central China. *Quat. Int.* **7–8**, 91–95 (1990).

48. A. Pullen, P. Kapp, A. T. McCallister, H. Chang, G. E. Gehrels, C. N. Garzione, R. V. Heermance, L. Ding, Qaidam Basin and northern Tibetan Plateau as dust sources for the Chinese Loess Plateau and paleoclimatic implications. *Geology* **39**, 1031–1034 (2011).

49. X. Liu, H. Dong, X. Yang, U. Herzschuh, E. Zhang, J.-B. W. Stuut, Y. Wang, Late Holocene forcing of the Asian winter and summer monsoon as evidenced by proxy records from the northern Qinghai–Tibetan Plateau. *Earth Planet. Sci. Lett.* **280**, 276–284 (2009).

50. F. Chen, Z. Yu, M. Yang, E. Ito, S. Wang, D. B. Madsen, X. Huang, Y. Zhao, T. Sato, H. John, B. Birks, I. Boomer, J. Chen, C. An, B. Wünnemann, Holocene moisture evolution in arid

central Asia and its out-of-phase relationship with Asian monsoon history. *Quat. Sci. Rev.* **27**, 351–364 (2008).

51. K. Nagashima, R. Tada, A. Tani, S. Toyoda, Y. Sun, Y. Isozaki, Contribution of aeolian dust in Japan Sea sediments estimated from ESR signal intensity and crystallinity of quartz. *Geochem. Geophys. Geosyst.* **8**, 10.1029/2006GC001364 (2007).

52. K. Nagashima, R. Tada, Teleconnection mechanism between millennial-scale Asian Monsoon dynamics and North Atlantic climate. *PAGES News* **20**, 64–65 (2012).

53. S. K. Marx, B. S. Kamber, H. A. McGowan, L. M. Petherick, G. H. McTainsh, N. Stromsoe, J. N. Hooper, J.-H. May, Palaeo-dust records: A window to understanding past environments. *Global Planet. Change* **165**, 13–43 (2018).

54. X. Liu, Z.-Y. Yin, X. Zhang, X. Yang, Analyses of the spring dust storm frequency of northern China in relation to antecedent and concurrent wind, precipitation, vegetation, and soil moisture conditions. *J. Geophys. Res. Atmos.* **109**, D16210 (2004).

55. J. Guo, H. Xu, L. Liu, D. Chen, Y. Peng, S. H.-L. Yim, Y. Yang, J. Li, C. Zhao, P. Zhai, The trend reversal of dust aerosol over East Asia and the North Pacific Ocean attributed to large-scale meteorology, deposition, and soil moisture. *J. Geophys. Res. Atmos.* **124**, 10450–10466 (2019).

56. P. Zhou, Z. Shi, X. Li, W. Zhou, Response of westerly jet over the Northern Hemisphere to astronomical insolation during the Holocene. *Front. Earth Sci.* **8**, 282 (2020).

57. S. Kaboth-Bahr, A. Bahr, C. Zeeden, K. A. Yamoah, M. A. Lone, C.-K. Chuang, L. Löwemark, K.-Y. Wei, A tale of shifting relations: East Asian summer and winter monsoon variability during the Holocene. *Sci. Rep.* **11**, 6938 (2021).

58. A. L. Hillman, M. B. Abbott, J. Yu, D. J. Bain, T. Chiou-Peng, Environmental legacy of copper metallurgy and Mongol silver smelting recorded in Yunnan lake sediments. *Environ. Sci. Technol.* **49**, 3349–3357 (2015).

59. A. L. Hillman, J. Yu, M. B. Abbott, C. A. Cooke, D. J. Bain, B. A. Steinman, Rapid environmental change during dynastic transitions in Yunnan Province, China. *Quat. Sci. Rev.* **98**, 24–32 (2014).

60. S. Wu, Y. Wei, B. Head, Y. Zhao, S. Hanna, The development of ancient Chinese agricultural and water technology from 8000 BC to 1911 AD. *Palgrave Commun.* **5**, 77 (2019).

61. L. Brandt, D. Ma, T. G. Rawski, "Industrialization in China" in *The Spread of Modern Industry to the Periphery Since 1871*, K. H. O'Rourke, J. G. Williamson, Eds. (Oxford Univ. Press, 2017), pp. 197–228.

62. D. H. Perkins, J. P. Tang, "East Asian industrial pioneers: Japan, Korea, and Taiwan" in *The Spread of Modern Industry to the Periphery Since 1871*, K. H. O'Rourke, J. G. Williamson, Eds. (Oxford Univ. Press, 2017), pp. 169–196.

63. J. Kurokawa, T. Ohara, Long-term historical trends in air pollutant emissions in Asia: Regional Emission inventory in ASia (REAS) version 3. *Atmos. Chem. Phys.* **20**, 12761–12793 (2020).

64. J. Hooper, S. K. Marx, J.-H. May, L. C. Lupo, J. J. Kulemeyer, E. de los Á, Pereira, O. Seki, H. Heijnis, D. Child, P. Gadd, A. Zawadzki, Dust deposition tracks late-Holocene shifts in monsoon activity and the increasing role of human disturbance in the Puna-Altiplano, northwest Argentina. *Holocene* **30**, 519–536 (2020).

65. J. Zhang, China's success in increasing per capita food production. *J. Exp. Bot.* **62**, 3707–3711 (2011).

66. X. Deng, J. Huang, S. Rozelle, E. Uchida, Cultivated land conversion and potential agricultural productivity in China. *Land Use Policy* **23**, 372–384 (2006).

67. W. Ta, Z. Dong, C. Sanzhi, Effect of the 1950s large-scale migration for land reclamation on spring dust storms in Northwest China. *Atmos. Environ.* **40**, 5815–5823 (2006).

68. X. Deng, Y. Luo, S. Dong, X. Yang, Impact of resources and technology on farm production in northwestern China. *Agr. Syst.* **84**, 155–169 (2005).

69. I. N. Sokolik, K. Darmenova, J. Huang, O. Kalashnikova, Y. Kurosaki, X. Xi, "Chapter 9. Examining changes in land cover and land use, regional climate and dust in dryland East Asia and their linkages within the Earth system" in *Dryland East Asia: Land Dynamics Amid Social and Climate Change*, C. Jiquan, W. Shiqiang, H. Geoffrey, Q. Jiaguo, G. Garik, S. Ge, K. Martin, Eds. (De Gruyter, 2013), pp. 183–212.

70. F. R. Li, L. F. Kang, H. Zhang, L. Y. Zhao, Y. Shirato, I. Taniyama, Changes in intensity of wind erosion at different stages of degradation development in grasslands of Inner Mongolia, China. *J. Arid Environ.* **62**, 567–585 (2005).

71. D. S. Hamilton, M. M. G. Perron, T. C. Bond, A. R. Bowie, R. R. Buchholz, C. Guieu, A. Ito, W. Maenhaut, S. Myriokefalitakis, N. Olgun, S. D. Rathod, K. Schepanski, A. Tagliabue, R. Wagner, N. M. Mahowald, Earth, wind, fire, and pollution: Aerosol nutrient sources and impacts on ocean biogeochemistry. *Ann. Rev. Mar. Sci.* **14**, 303–330 (2022).

72. L. Li, S. Bai, J. Li, S. Wang, L. Tang, S. Dasgupta, Y. Tang, X. Peng, Volcanic ash inputs enhance the deep-sea seabed metal-biogeochemical cycle: A case study in the Yap Trench, western Pacific Ocean. *Mar. Geol.* **430**, 106340 (2020).

73. S. Duggen, N. Olgun, P. Croot, L. Hoffmann, H. Dietze, P. Delmelle, C. Teschner, The role of airborne volcanic ash for the surface ocean biogeochemical iron-cycle: A review. *Biogeosciences* **7**, 827–844 (2010).

74. D. Fink, Q. Hotchkis, Q. Hua, G. Jacobsen, A. M. Smith, U. Zooppi, D. M. Child, C., H. van der Gaast, A. Williams, M. Willima, The ANTARES facility at ANSTO. *Nucl. Instrum. Methods Phys. Res. B Beam Interact. Mater. At.* **223–224**, 109–115 (2004).

75. Q. Hua, G. E. Jacobson, U. Zoppi, E. M. Lawson, A. A. Williams, A. M. Smith, M. J. McGann, Progress in radiocarbon target preparation at the ANTARES AMS centre. *Radiocarbon* **43**, 275–282 (2001).

76. S. K. Marx, B. S. Kamber, H. A. McGowan, Estimates of Australian dust flux into New Zealand: Quantifying the eastern Australian dust plume pathway using trace element calibrated ^{210}Pb as a monitor. *Earth Planet. Sci. Lett.* **239**, 336–351 (2005).

77. S. K. Marx, J. M. Knight, P. G. Dwyer, D. P. Child, M. A. C. Hotchkis, A. Zawadzki, Examining the response of an eastern Australian mangrove forest to changes in hydro-period over the last century. *Estuar. Coast. Shelf Sci.* **241**, 106813 (2020).

78. S. M. Eggins, J. D. Woodhead, L. P. J. Kinsley, G. E. Mortimer, M. T. Slyvester, M. T. McCulloch, J. M. Hergt, M. R. Handler, A simple method for the precise determination of ≥40 trace elements in geological samples by ICPMS using enriched isotope internal standardisation. *Chem. Geol.* **134**, 311–326 (1997).

79. B. S. Kamber, Geochemical fingerprinting: 40 years of analytical development and real world applications. *Appl. Geochem.* **24**, 1074–1086 (2009).

80. A. Retzmann, T. Zimmermann, D. Pröfrock, T. Prohaska, J. Irrgeher, A fully automated simultaneous single-stage separation of Sr, Pb, and Nd using DGA Resin for the isotopic analysis of marine sediments. *Anal. Bioanal. Chem.* **409**, 5463–5480 (2017).

81. P. J. Reimer, W. E. N. Austin, E. Bard, A. Bayliss, P. G. Blackwell, C. Bronk Ramsey, M. Butzin, H. Cheng, R. L. Edwards, M. Friedrich, P. M. Grootes, T. P. Guilderson, I. Hajdas, T. J. Heaton, A. G. Hogg, K. A. Hughen, B. Kromer, S. W. Manning, R. Muscheler, J. G. Palmer, C. Pearson, J. van der Plicht, R. W. Reimer, D. A. Richards, E. M. Scott, J. R. Southon, C. S. M. Turney, L. Wacker, F. Adolphi, U. Büntgen, M. Capano, S. M. Fahrni, A. Fogtmann-Schulz, R. Friedrich, P. Köhler, S. Kudsk, F. Miyake, J. Olsen, F. Reinig, M. Sakamoto, A. Sookdeo, S. Talamo, The IntCal20 Northern Hemisphere radiocarbon age calibration curve (0–55 cal kBP). *Radiocarbon* **62**, 725–757 (2020).

82. Q. Hua, J. C. Turnbull, G. M. Santos, A. Z. Rakowski, S. Ancapichún, R. De Pol-Holz, S. Hammer, S. J. Lehman, I. Levin, J. B. Miller, J. G. Palmer, C. S. M. Turney, Atmospheric radiocarbon for the period 1950-2019. *Radiocarbon* **64**, 723–745 (2022).

83. J. Lindeløv, mcp: An R package for regression with multiple change points (2020).

84. A. Parnell, simmr: A stable isotope mixing model (2021); https://CRAN.R-project.org/package=simmr.

85. E. Govan, A. L. Jackson, R. Inger, S. Bearhop, A. C. Parnell, simmr: A package for fitting stable isotope mixing models in R. arXiv:2306.07817 [stat.AP] (2023).

86. A. Bouvier, J. D. Vervoort, P. J. Patchett, The Lu–Hf and Sm–Nd isotopic composition of CHUR: Constraints from unequilibrated chondrites and implications for the bulk composition of terrestrial planets. *Earth Planet. Sci. Lett.* **273**, 48–57 (2008).

87. M. A. Aquino-López, M. Blaauw, J. A. Christen, N. K. Sanderson, Bayesian analysis of ^{210}Pb dating. *J. Agric. Biol. Environ. Stat.* **23**, 317–333 (2018).

88. A. L. Heathwaite, K. H. Göttlich, E. G. Burmeister, G. Kaule, T. H. Grospietsch, "Mires: Definitions and form" in *Mires: Processes*, *Exploitation and Conservation*, A. L. Heathwaite, K. H. Göttlich, Eds. (John Wiley & Sons, 1990), pp. 1–75.

89. S. K. Marx, B. S. Kamber, H. McGowan, A.,, J. Denholm, Holocene dust deposition rates in Australia's Murray-Darling Basin record the interplay between aridity and the position of the mid-latitude westerlies. *Quat. Sci. Rev.* **30**, 3290–3305 (2011).

90. S. Pratte, K. Bao, A. Sapkota, W. Zhang, J. Shen, G. Le Roux, F. De Vleeschouwer, 14 kyr of atmospheric mineral dust deposition in north-eastern China: A record of palaeoclimatic and palaeoenvironmental changes in the Chinese dust source regions. *Holocene* **30**, 492–506 (2019).

91. G. Le Roux, W. Shotyk, "Chapter 9 Weathering of inorganic matter in bogs" in *Developments in Earth Surface Processes*, I. P. Martini, A. Martínez Cortizas, W. Chesworth, Eds. (Elsevier, 2006), vol. 9, pp. 197–215.

92. A. Sapkota, A. K. Cheburkin, G. Bonani, W. Shotyk, Six millennia of atmospheric dust deposition in southern South America (Isla Navarino, Chile). *Holocene* **17**, 561–572 (2007).

93. W. Shotyk, D. Weiss, P. G. Appleby, A. Cheburkin, M. Gloor, J. D. Kramers, S. Reese, W. O. Van Der Knaap, History of atmospheric lead deposition since 12,370 14C yr BP from a peat bog, Jura Mountains, Switzerland. *Science* **281**, 1635–1640 (1998).

94. K. T. Tolonen, Interpretation of changes in the ash content of ombrotrophic peat layers. *Bull. Geol. Soc. Finl.* **56**, 207–219 (1984).

95. D. Weiss, W. Shotyk, E. A. Boyle, J. D. Kramers, P. G. Appleby, A. K. Cheburkin, Comparative study of the temporal evolution of atmospheric lead deposition in Scotland and eastern Canada using blanket peat bogs. *Sci. Total Environ.* **292**, 7–18 (2002).

96. K. P. Jochum, U. Weis, B. Schwager, B. Stoll, S. A. Wilson, G. H. Haug, M. O. Andreae, J. Enzweiler, Reference values following ISO guidelines for frequently requested rock reference materials. *Geostand. Geoanal. Res.* **40**, 333–350 (2016).

97. D. Weis, B. Kieffer, C. Maerschalk, J. Barling, J. de Jong, G. A. Williams, D. Hanano, W. Pretorius, N. Mattielli, J. S. Scoates, A. Goolaerts, R. M. Friedman, J. B. Mahoney, High-precision isotopic characterization of USGS reference materials by TIMS and MC-ICP-MS. *Geochem. Geophys. Geosyst.* **7**, 10.1029/2006GC001283 (2006).

98. T. Tanaka, S. Togashi, H. Kamioka, H. Amakawa, H. Kagami, T. Hamamoto, M. Yuhara, Y. Orihashi, S. Yoneda, H. Shimizu, T. Kunimaru, K. Takahashi, T. Yanagi, T. Nakano, H. Fujimaki, R. Shinjo, Y. Asahara, M. Tanimizu, C. Dragusanu, JNdi-1: A neodymium isotopic reference in consistency with LaJolla neodymium. *Chem. Geol.* **168**, 279–281 (2000).
